# Modelling Blood Flow and Metabolism in the Preclinical Neonatal Brain during and Following Hypoxic-Ischaemia

**DOI:** 10.1371/journal.pone.0140171

**Published:** 2015-10-07

**Authors:** Matthew Caldwell, Tracy Moroz, Tharindi Hapuarachchi, Alan Bainbridge, Nicola J. Robertson, Chris E. Cooper, Ilias Tachtsidis

**Affiliations:** 1 Medical Physics and Biomedical Engineering, University College London, London, United Kingdom; 2 CoMPLEX, University College London, London, United Kingdom; 3 Medical Physics and Bioengineering, UCLH NHS Foundation Trust, London, United Kingdom; 4 Insititute for Women’s Health, University College London, London, United Kingdom; 5 Biological Sciences, University of Essex, Colchester, United Kingdom; Hungarian Academy of Sciences, HUNGARY

## Abstract

Hypoxia-ischaemia (HI) is a major cause of neonatal brain injury, often leading to long-term damage or death. In order to improve understanding and test new treatments, piglets are used as preclinical models for human neonates. We have extended an earlier computational model of piglet cerebral physiology for application to multimodal experimental data recorded during episodes of induced HI. The data include monitoring with near-infrared spectroscopy (NIRS) and magnetic resonance spectroscopy (MRS), and the model simulates the circulatory and metabolic processes that give rise to the measured signals. Model extensions include simulation of the carotid arterial occlusion used to induce HI, inclusion of cytoplasmic pH, and loss of metabolic function due to cell death. Model behaviour is compared to data from two piglets, one of which recovered following HI while the other did not. Behaviourally-important model parameters are identified via sensitivity analysis, and these are optimised to simulate the experimental data. For the non-recovering piglet, we investigate several state changes that might explain why some MRS and NIRS signals do not return to their baseline values following the HI insult. We discover that the model can explain this failure better when we include, among other factors such as mitochondrial uncoupling and poor cerebral blood flow restoration, the death of around 40% of the brain tissue.

## Introduction

Neonatal hypoxia-ischaemia (HI) is a major cause of brain injury in term infants. In developed countries, its incidence is 1 to 2 per 1000 live births, and it is estimated to account for 23% of worldwide neonatal deaths [1]. HI leads to long term neurological problems in up to 25% of survivors [2] including cerebral palsy and epilepsy [3]. Monitoring and early detection of cerebral circulatory and metabolic disturbances are very important for assessment of brain injury, in addition to the development and timely application of neuroprotective strategies such as hypothermia [4]. Understanding the time evolution of changes in brain oxygenation, haemodynamics and metabolism during and following HI is a highly active area of research that often involves multimodal monitoring with advanced techniques and technologies. Integrative, multiscale computational models of the brain can assist the interpretation of such monitoring and provide insights into the physiological and biochemical processes involved.

Non-invasive monitoring of brain physiology and biochemistry is extremely challenging. The current state-of-the-art techniques for human infants and piglets (a preclinical animal model of human neonates) are broadband near-infrared spectroscopy (NIRS) [5, 6] and magnetic resonance spectroscopy (MRS) [7–9].

Broadband NIRS uses multi-wavelength near-infrared light to measure tissue concentration changes of oxy- and deoxy-haemoglobin (ΔHbO_2_ and ΔHHb). It can also be used to monitor changes in the oxidation state of cytochrome c oxidase (CCO), the terminal acceptor in the electron transport chain. CCO is located in the mitochondrial membrane, and passes electrons to oxygen to form water. Changes in oxidative metabolism can lead to changes in the redox state of CCO. NIRS can be used to measure the change in concentration of oxidised CCO (ΔoxCCO) which is indicative of the redox state of CCO. Changes in ΔoxCCO have been observed in response to changes in inspired oxygen in a variety of species [10–12].

MRS can measure the concentration of various metabolites in tissue, depending on which type of MRS is used. ^31^P-MRS measures concentrations of the phosphorus-containing metabolites adenosine triphosphate (ATP), phosphocreatine (PCr) and inorganic phosphate (P_i_). The spectrum can also be used to calculate pH from the chemical shifts of certain peaks [13]. MRS measurements are often expressed as ratios because this avoids the difficulties of determining absolute concentrations.

NIRS and MRS are complementary techniques that we have been using together for several years to investigate HI in the piglet [10, 14]. The brain physiology and biochemistry of the piglets can be monitored with both modalities throughout the insult, recovery and treatment. In a recently-published study, combining broadband NIRS and ^31^P-MRS during and after hypoxic-ischaemia in 24 new born piglets [15], we found significant correlations between brain tissue changes in [oxCCO] and those of PCr, P_i_ and nucleotide triphosphate (NTP, mainly ATP). These correlations were not reflected in the haemoglobin signals. We further demonstrated that following HI the recovery fraction of the broadband NIRS measurement of [oxCCO] was highly correlated with the recovery fraction of the ^31^P-MRS measurement of NTP and outcome at 48h.

We are currently working towards interpreting the relationships between the measurements from the two modalities, and investigating the possibility of combining them to give a better picture of the health of the brain following perinatal asphyxia. To help with this, we have developed a multiscale computational model to simulate HI, and the NIRS and MRS signals arising from it, in the neonatal piglet brain. The model is based on a representation of the underlying brain tissue physiology and biochemistry. It can be used to combine measurements from these modalities, helping to uncover the complex and non-linear relationships between them and investigate their physiological consequences.

Computational modelling has frequently been applied both to cerebral circulation [16–18] and to oxidative metabolism in the brain and other tissues [19–21]. In recent years, there have been a number of models representing oxygen delivery and metabolism in the brain. The model of Aubert et al. [22] in particular has been modified and extended in several ways to model different aspects of cerebral metabolism, including astrocyte and neuron interaction [23, 24] and pH changes [25].

Modelling of brain circulation and metabolism has been carried out in our group since 2005 [26]. The earliest model (BrainCirc) was created to investigate autoregulation, and includes equations representing blood flow, ion channel activity in the vascular smooth muscle and respiration from glycolysis to the electron transport chain. The circulatory portion of the model was derived from that of Ursino and Lodi [16]. A second model, BrainSignals, was developed in 2008, primarily to simulate NIRS signals [27]. BrainSignals is intentionally simpler and more comprehensible than BrainCirc, but models the electron transport chain in more detail in order to simulate the ΔoxCCO signal. BrainSignals has been validated with both *in vivo* and *in vitro* data, and its outputs compared with measurements from hypoxia challenges [28], hypercapnia challenges [29] and anagram solving tasks in healthy adults [30]. The BrainSignals model was subsequently adapted from adult human to neonatal piglet physiology and expanded to simulate signals measured by MRS. The resulting BrainPiglet model has been applied to averaged data from brief anoxia in piglets [31].

Most complex, multiscale computational models depend on a large number of parameters to tune the model behaviour. These may represent real physical quantities or convenient abstractions. Some may be known with great precision, but typically many will be quite uncertain and some may be impossible to measure at all. Investigating how a model’s behaviour depends on its parameters—its sensitivity to them—can therefore be important in interpreting its outputs [32, 33]. A property common to many such models is what has been termed ‘sloppiness’ [34, 35]: a high-dimensional parameter space that interpolates a smaller space of behavioural variation, with a roughly exponential distribution of sensitivities. Only a few directions in parameter space, termed ‘stiff’, have a strong effect on behaviour, while most parameters are ‘sloppy’, affecting behaviour jointly but not uniquely identified by it. Fitting sloppy models may give parameter estimates that are wildly inaccurate whilst still being meaningfully predictive of behaviour.

In this paper we present a significantly expanded version of the BrainPiglet model, which we term BrainPigletHI. This model includes: (i) simulation of carotid artery occlusion, which is routinely used in piglets to induce HI; (ii) simulation of cytoplasmic pH, both as a participant in metabolic reactions and as a model output; and (iii) the ability to simulate cell death, in order to model poor physiological recovery following HI. A shift in brain pH is known to occur following HI [36], and changes in pH can alter protein structure and hence affect cell function. It is therefore important that pH is properly taken into account in our model. Here we apply the model to multimodal piglet data from our recent published experiments [15], and use it to investigate possible explanations for the non-recovery of one of the piglets. Our group has also applied the model to the simulation of grouped data from recovering piglets, with results reported in [37]. (In that report the model was not defined in detail, so we note here that it included cytoplasmic pH and occlusion, but did not consider non-recovery or cell death.)

## Methods

### Ethics Statement

All animal experiments were performed under UK Home Office Guidelines (Animals [Scientific Procedures] Act, 1986) under Project Licence Number PPL 70/7203, approved by the UCL Animal Welfare and Ethical Review Body (AWERB). All surgery was performed under anaesthesia, and every effort was made to minimize suffering.

### The BrainPigletHI Model

**Fig 1:**
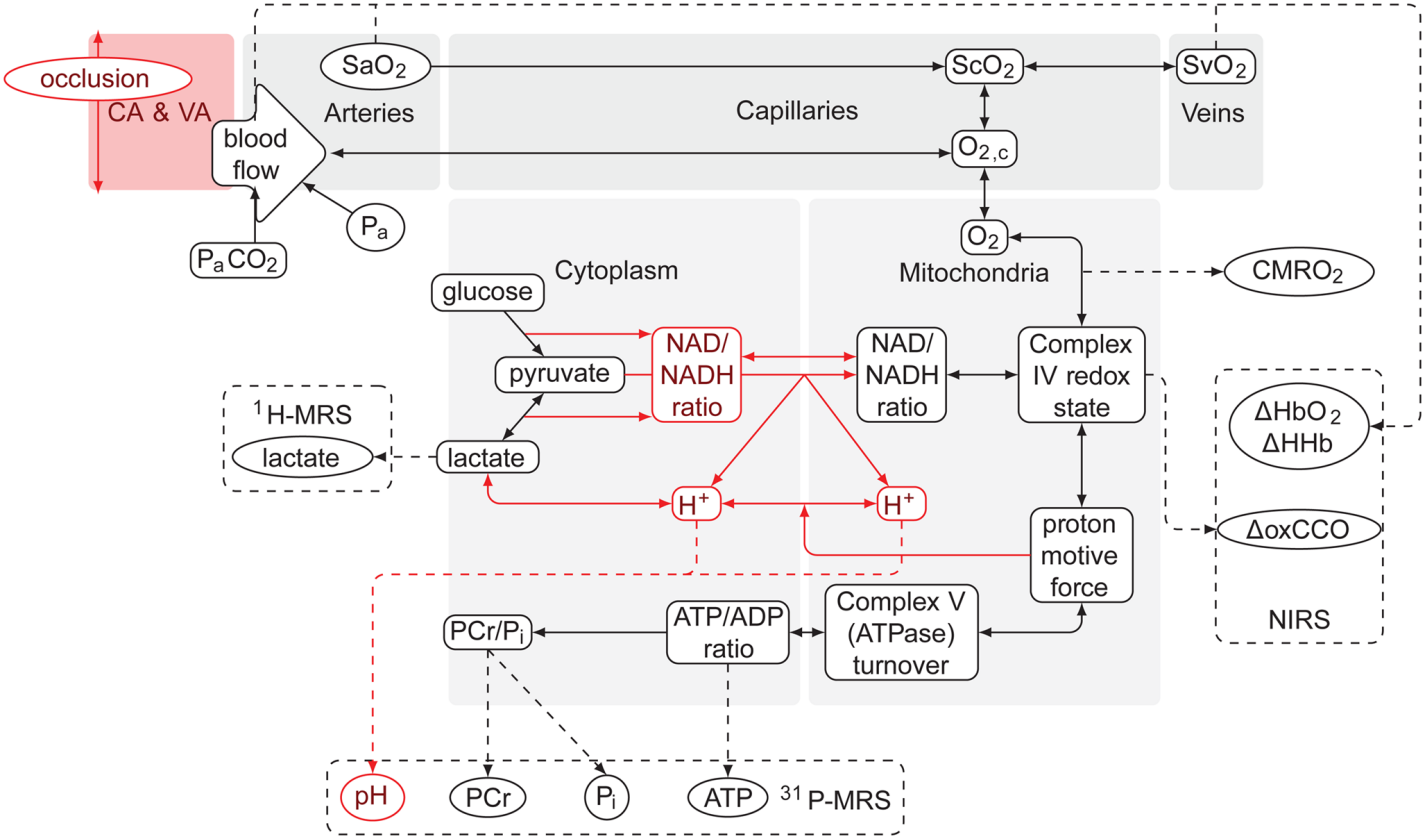
Overall structure of the BrainPigletHI model. Major changes to the original BrainPiglet are indicated in red. Blood flows from the carotid and other supplying arteries, through the cerebral arteries to the capillaries, exiting via the veins. Oxygen dissociates from haemoglobin in the capillaries and diffuses to the tissue, where it participates in the final step of the mitochondrial electron transport chain. Metabolic reactions in the cytoplasmic compartment affect substrate supply and pH. Dashed lines indicate model outputs that may be compared with measurements from NIRS and MRS.

BrainPigletHI is an extension of the previously-published BrainPiglet model [31], describing circulation and cerebral metabolism in the neonatal piglet brain. The compartments and main processes of the model are illustrated schematically in Fig 1, with the changes indicated in red.

The model changes comprise three distinct areas, each of which has a bearing on the understanding of HI: (i) modelling of the carotid occlusion that is used in experimental models of HI; (ii) modelling cytoplasmic pH, which affects the metabolic processes during HI, and is an important contributor to a measurable signal; and (iii) a representation of cell death, which is a possible explanation for the non-recovery observed in some experimental data.

The overall model structure is the same for both BrainPiglet and BrainPigletHI. A circulatory part models the cerebral blood flow (CBF) through a series of vascular compartments, while a metabolic part models intracellular chemical reactions in the cytoplasm and mitochondria. Blood flow is regulated in response to several factors, including the capillary oxygen concentration and the mean arterial blood pressure, by modulation of the vessel radius in the cerebral arterial compartment. The metabolic processes depend on oxygen delivery from the blood. A number of output variables are predicted from the modelled physiological and biochemical state, which may be compared with values measured by NIRS and ^31^P-MRS, as well as other modalities not measured in this study, such as ^1^H-MRS, which is often used clinically to measure the brain tissue lactate levels in hypoxic-ischaemic infants; and transcranial Doppler, which can measure the velocity of the middle cerebral artery, an indicator of cerebral blood flow.

In the circulatory part of the model, the blood flow through a compartment depends on the compartment conductance and the pressure difference across it. The standard analogy is to electrical circuits, with the relationship equivalent to Ohm’s Law. (Vessel compliance is not included in BrainPigletHI, but it it were there would be a corresponding analogy to capacitance.) For example, in the cerebral arterial compartment the conductance is denoted G, the pressure at the supply side is P_a2_ and the pressure at the venous side is P_v_, so the blood flow is given by

$$\begin{array}{c} CBF=G(P_{a2}-P_{v}) \end{array}$$ (1)

**Fig 2:**
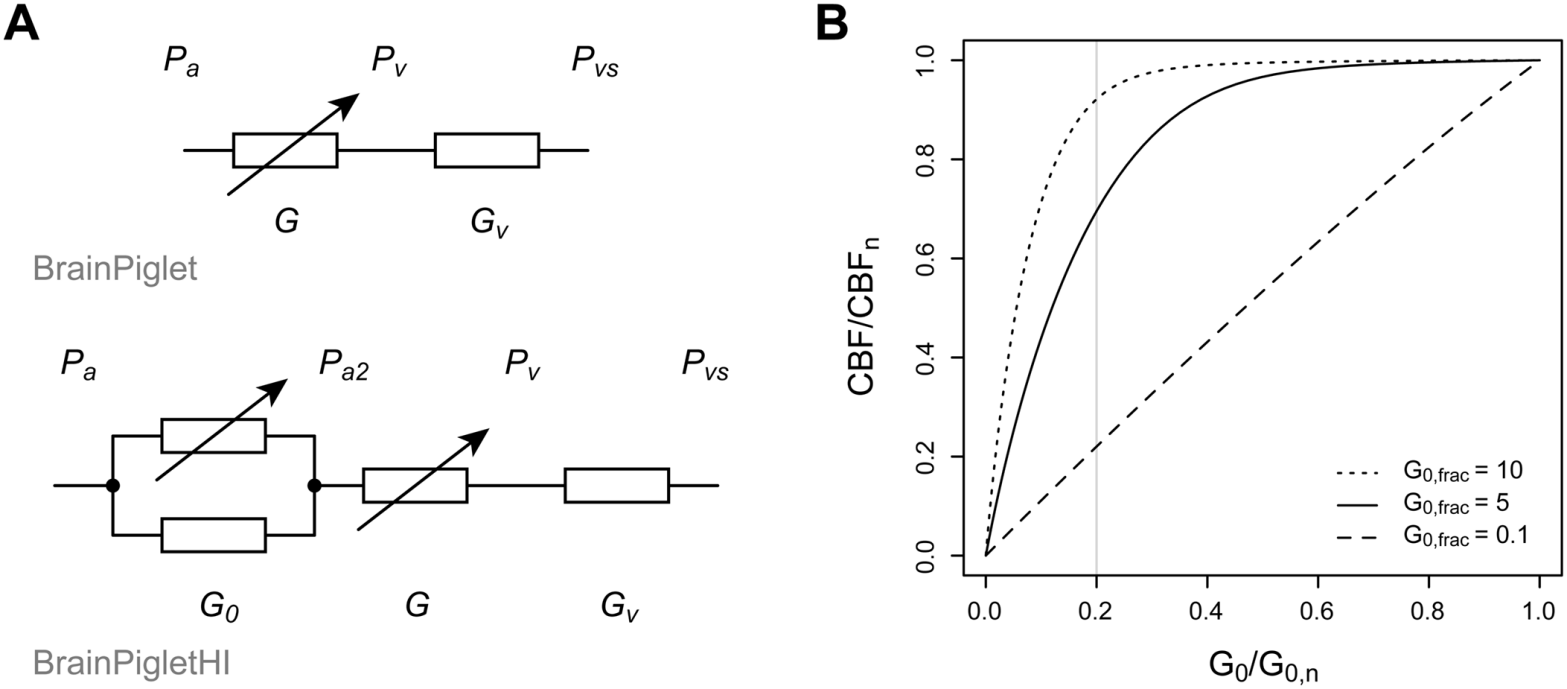
Changes to the circulatory part of the model. (A) Electrical circuit analogues of the blood flow components of BrainPiglet and BrainPigletHI. A new compartment has been added to the latter, with total conductance G_0_, to represent supplying arteries. The portion of this conductance that is through the carotid arteries (G_0,n_occ_frac_) is shown as a variable resistor to indicate the possibility of experimental occlusion. (In both models the resistance of the capillary bed is assumed to be negligible.) (B) Relationship between cerebral blood flow and the relative conductance of the supplying arterial compartment. The curvature depends on the value of the parameter G_0,frac_. Examples are shown for G_0,frac_ = 10 (dotted), G_0,frac_ = 5 (solid) and G_0,frac_ = 0.1 (dashed). Complete occlusion of the carotid arteries is equivalent to reducing G_0_ to 20% of its normal value, marked by a grey line on the plot.

In order to allow simulation of carotid artery occlusion, a new compartment was added to the circulatory part of the model. The changes are illustrated in electrical circuit analogue form in Fig 2A. The original model included only one arterial-arteriolar compartment, with conductance G under autoregulatory control. This compartment remains in BrainPigletHI, but is now preceded by another compartment representing the supplying arteries, with conductance G_0_. The latter is not subject to autoregulatory control, and under normal conditions is assumed to have a constant value G_0,n_. However, when the carotid arteries are occluded experimentally, the conductance is reduced.

The carotid arteries are responsible for the majority of the cerebral blood supply: 80% in adult humans under normal conditions [38]. In the model, this fraction is denoted occ_frac_. Hence, if these arteries were completely occluded, the conductance would be reduced to

$$\begin{array}{c} G_{0}=G_{0,n}\left(1-occ_{frac}\right) \end{array}$$ (2)

In practice, it is useful to be able to model an incomplete or gradual occlusion, so an additional control parameter is included, k_occ_, to specify the degree of occlusion:

$$\begin{array}{c} G_{0}=G_{0,n}\left(1-k_{occ}occ_{frac}\right) \end{array}$$ (3)

The normal, unoccluded conductance of the supplying compartment is set relative to the normal value for the cerebral arterial compartment, G_n_:

$$\begin{array}{c} G_{0,n}=G_{0,frac}G_{n} \end{array}$$ (4)

The weighting factor G_0,frac_ expresses the relative contribution of the two compartments to the flow resistance. This cannot be readily measured, so instead it is set by considering the curve of CBF as a function of G_0_ at steady state (Fig 2B). Intuitively, we would expect the larger supplying vessels to offer less resistance to blood flow than the smaller cerebral vessels, corresponding to a high value for G_0,frac_. If G_0,frac_ were lower (signifying a higher resistance in the supplying arteries), then CBF would be more rapidly constrained by occlusion of the supply. A few studies have reported CBF measurements in piglets when one or both carotid arteries were occluded but oxygen levels were normal. The occlusion of just one carotid artery did not significantly reduce CBF. With both carotid arteries occluded, measurements of CBF include 75% [39] and 45% [40] of baseline values; however, these experiments also involved changes in systemic arterial blood pressure. For our purposes, a value of G_0,frac_ = 5.0 was chosen so that the curve lay between these two values.

In BrainPiglet, the pressure at the start of the cerebral arterial compartment is assumed to be the systemic arterial pressure, P_a_. In BrainPigletHI this is no longer the case, since there is a pressure drop across the supplying compartment. The new boundary pressure, P_a2_, is calculated by equating the blood flow through the two compartments:

$$\begin{array}{c} G_{0}\left(P_{a}-P_{a2}\right)=G\left(P_{a2}-P_{v}\right) \end{array}$$ (5)

where P_v_ is the pressure at the start of the venous compartment. (This is equivalent to calculating the voltage drop across the series resistances in the electrical circuit of Fig 2A.)

The second addition to the model was the simulation of cytoplasmic pH. Protons are involved in metabolic reactions in both cytoplasm and mitochondria, but only mitochondrial pH was modelled explicitly in BrainPiglet. Inclusion of explicit cytoplasmic pH should improve the behavioural fidelity of the model. Moreover, intracellular pH can be inferred from ^31^P-MRS spectra [13], but the measurement is not localised to a single compartment. Since the mitochondria constitute only a tiny fraction of the tissue volume, the cytoplasmic pH is likely to dominate the aggregate measurement. Without explicit modelling of that value, we cannot make a meaningful comparison to the data.

**Fig 3:**
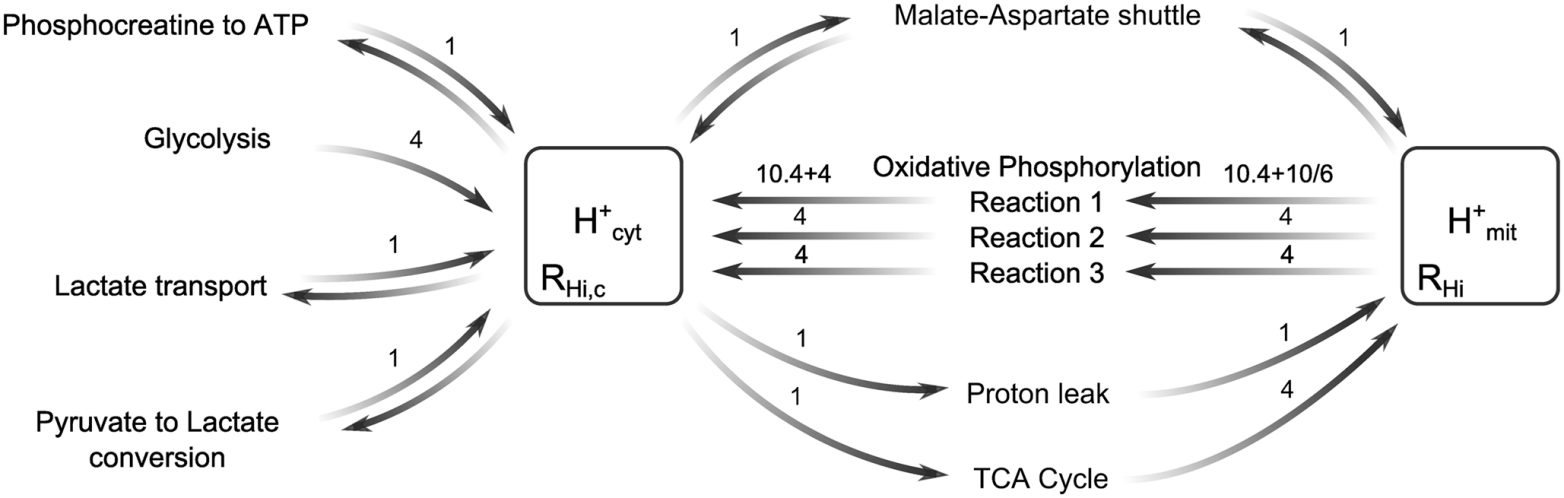
Cytoplasmic and mitochondrial reactions extended to include explicit proton changes. Buffering is taken into account by defining effective volumes of the compartments for protons (R_Hi_ and R_Hi,c_) as described in Eq (9). The model equation for the ATP synthesis reaction also takes into account proton leak. The fractional number of protons in reaction 1 arises from a weighted average of reduction from both NADH and FADH_2_.

Protons were added to the model’s oxidative phosphorylation reactions, the TCA cycle, glycolysis, pyruvate to lactate conversion, lactate transport, and the phosphocreatine equilibrium. In addition, cytoplasmic NAD/NADH was added to the model, and included in glycolysis and the reaction describing the conversion between pyruvate and lactate. The network of reactions involving protons in the cytoplasm and mitochondria is illustrated in Fig 3.

The rates of the oxidative phosphorylation reactions and the TCA cycle were left unchanged. Glycolysis is modelled in BrainPiglet as a Michaelis-Menten reaction. Its rate term was updated to include a dependence on NAD concentration:

$$\begin{array}{c} \frac{v_{glyc}{\left[\text{ADP}\right]}^{2}{\left[P_{i}\right]}^{2}\left[\text{gluc}\right]{\left[{\text{NAD}}_{cyt}\right]}^{2}}{\left(k_{m,glyc_{A}}^{2}+{\left[\text{ATP}\right]}^{2}\right)\left(k_{m,glyc_{P}}^{2}+{\left[P_{i}\right]}^{2}\right)\left(k_{m,glyc_{G}}+\left[\text{gluc}\right]\right)\left(k_{m,glyc_{N}}^{2}+{\left[{\text{NAD}}_{cyt}\right]}^{2}\right)} \end{array}$$ (6)

The k_m_ are rate constants relating to each reactant and v_glyc_ is the maximum rate for glycolysis.

As in BrainPiglet, lactate transport into and out of the cell is considered as the balance of two symmetrical reactions, each with a maximum rate v_MCT_, dependent on the lactate concentration on the relevant side of the cell membrane. Lactate is co-transported with a proton, and the transport rate is known to be somewhat affected by pH [41]. BrainPigletHI includes support for this dependence via additional rate terms in the two transport reactions:

$$\begin{array}{c} v_{MCT}(\frac{\left[{\text{lac}}_{cap}\right]\left[H_{cap}^{+}\right]}{\left(k_{MCT}+\left[{\text{lac}}_{cap}\right]\right)\left(k_{MCT,H}+\left[H_{cap}^{+}\right]\right)}-\frac{\left[\text{lac}\right]\left[H_{cyt}^{+}\right]}{\left(k_{MCT}+\left[\text{lac}\right]\right)\left(k_{MCT,H}+\left[H_{cyt}^{+}\right]\right)}) \end{array}$$ (7)

The capillary concentrations, [H_cap_^+^] and [lac_cap_] are assumed constant, while k_MCT_ and k_MCT,H_ are rate constants for the two substrates. However, inclusion of this pH dependency produced a negligible effect unless k_MCT,H_ was given an unrealistic value, in which case the results were also unrealistic, so for practical purposes this parameter was set to 0 and the net transport rate simplified to its original BrainPiglet version.

All the other reactions are modelled as mass action reactions, and their rates were updated accordingly to include the concentration of hydrogen ions.

The Malate Aspartate shuttle, which allows the exchange of NAD and NADH between the cytoplasm and mitochondria, was added to the model as a mass action reaction:

$$\begin{array}{c} {\text{NADH}}_{cyt}+{\text{NAD}}_{mit}+H_{cyt}^{+}⇌{\text{NADH}}_{mit}+{\text{NAD}}_{cyt}+H_{mit}^{+} \end{array}$$ (8)

The equilibrium constant for this reaction was set to 10 [26] and the rate constants were chosen to give a steady state at the normal value of CMRO_2_.

BrainPiglet models proton buffering in the mitochondria by defining an effective mitochondrial volume for protons [19, 27]. The new model extends this approach to the cytoplasm, defining an equivalent cytoplasmic effective volume:

$$\begin{array}{c} R_{Hi,c}=\frac{C_{buffi,c}}{(10^{-pH_{c}}-10^{-pH_{c}-dpH})/dpH}Vol_{cyt} \end{array}$$ (9)

Here, Vol_cyt_ is the real cytoplasmic volume, while C_buffi,c_ and dpH are constants. The latter is the same as for the mitochondrial buffering relationship.

As discussed below, several possible state changes were modelled to investigate piglet non-recovery following HI. Mitochondrial uncoupling is already represented in BrainPiglet by the parameter k_unc_, normally set to 1, a simple scale factor that multiplies the rate of proton entry to the mitochondrial matrix by mechanisms independent of ATP production, relative to its normal level [27]. Occlusion is represented via the parameter k_occ_, described above.

In order to simulate cell death, the model was extended as follows. A parameter d_f_ was added to represent the fraction of cells that have died. It was assumed that during HI, CCO becomes fully reduced and remains so in the dead cells. It was further assumed that in dead cells, all the exchangeable phosphate is in the form of P_i_.

In the model, oxygen is transferred from the capillaries to the mitochondria at a rate T_O_2_in_, dependent on the concentration gradient, and consumed at a rate T_box_. The time course of mitochondrial oxygen is determined by the interplay of these terms. Dead cells were assumed to no longer consume oxygen and quickly equilibrate with the oxygen concentration of the capillaries. Accordingly, the volume of functioning mitochondria, V_mit_, was scaled by (1–d_f_):

$$\begin{array}{c} \frac{d[O_{2}]}{dt}=\frac{T_{O_{2}in}}{V_{mit}\left(1-d_{f}\right)}-T_{box} \end{array}$$ (10)

Since concentration depends on volume, the rate of concentration change per unit of transferred oxygen increases as the living volume declines. This can be interpreted as reflecting increased oxygen availability as consumption falls. That in turn affects the concentration gradient, leading to reduced oxygen unbinding in the blood, ultimately reflected in the NIRS variables.

In addition, a number of model outputs were changed to account for the effect of cell death on measured quantities:

$$\begin{array}{c} \text{NTP}/\text{EPP}=\frac{\left(1-d_{f}\right)\left[\text{ATP}\right]}{\text{EPP}} \end{array}$$ (11)

$$\begin{array}{c} \text{PCr}/\text{EPP}=\frac{\left(1-d_{f}\right)\left[\text{PCr}\right]}{\text{EPP}} \end{array}$$ (12)

$$\begin{array}{c} P_{i}/\text{EPP}=\frac{\left(1-d_{f}\right)\left[P_{i}\right]}{\text{EPP}}+d_{f} \end{array}$$ (13)

$$\begin{array}{c} \Delta \text{oxCCO}=\left(1-d_{f}\right)\Delta \text{oxCCO}-d_{f}{\text{oxCCO}}_{n} \end{array}$$ (14)

$$\begin{array}{c} {\text{CMRO}}_{2}=\left(1-d_{f}\right){\text{CMRO}}_{2} \end{array}$$ (15)

EPP denotes the total exchangeable phosphate pool, given by [PCr] + [P_i_] + 2[NTP]. Note that the values calculated in Eqs (11)–(15) do not affect the model behaviour, only the predicted measurements arising from the model state.

All new model parameters and their values are listed in Table 1.

**Table 1:** New parameters in the BrainPigletHI model.

Parameter	Description	Value	Source
occ_frac_	Fraction of blood normally passing through carotid arteries	0.8	[38]
G_0,frac_	Ratio of normal conductances of the supplying arterial and cerebral arterial compartments	5	[39, 40]
C_buffi,c_	Constant in the cytoplasmic proton buffering relationship	10	[19]
K_eq,MAshut_	Equilibrium constant for the malate-aspartate shuttle	10	[26]
[NAD_cyt_]_n_	Normal concentration of NAD in the cytoplasm	359 mM	[42]
[NADH_cyt_]_n_	Normal concentration of NADH in the cytoplasm	50 mM	[42]
k_m,glycN_	Rate constant for NAD in the equation representing glycolysis	1 mM	[26]
k_MCT,H_	Rate constant for protons in the equation representing lactate transport	0 mM	–
d_f_	The fraction of dead cells following HI	0	–
k_occ_	Control parameter for the degree of carotid occlusion	0	–

### Experimental Methods

The model was applied to data from individual piglets which had been subjected to HI. The experimental methods have been described previously [43] but in brief, the piglets were mechanically ventilated and anaesthetised with isoflurane. Arterial oxygen saturation (SaO_2_), arterial blood pressure (P_a_) and heart rate were monitored continuously. Vascular occluders were placed around both common carotid arteries. Broadband NIRS data was acquired using an in house developed broadbrand spectrometer from optodes placed on either side of the head. Data were collected at 1 min intervals and the measurements of ΔHbO_2_, ΔHHb, and ΔoxCCO were determined from the attenuation spectra between 780 and 900 nm using the UCLn algorithm [44] after correcting for wavelength dependence of path-length [45]. The ^31^P-MR surface coil was placed and secured on top of the piglet head (without interfering with the NIRS optodes) and ^31^P-MRS spectra were acquired at 1 min intervals in a 9.4 T MR spectrometer. After 10 min of baseline measurements, HI was induced by occluding the carotid arteries and reducing the inspired oxygen fraction (FiO_2_) to 12%. It was held there until the height of the *β*-NTP peak had fallen to 50% of its baseline value. FiO_2_ was then adjusted to maintain the peak between 30 and 50% of its baseline height for 12.5 min, after which time the occluders were deflated and FiO_2_was returned to normal.

Intracellular pH was calculated from the ^31^P-MRS spectra following the methods described by Cady et al [13]. The chemical shift of P_i_ and phosphoethanolamine (PEt) was used to calculate an estimate of pH denoted pH_Pi–PEt_.

### Computational Simulations

**Fig 4:**
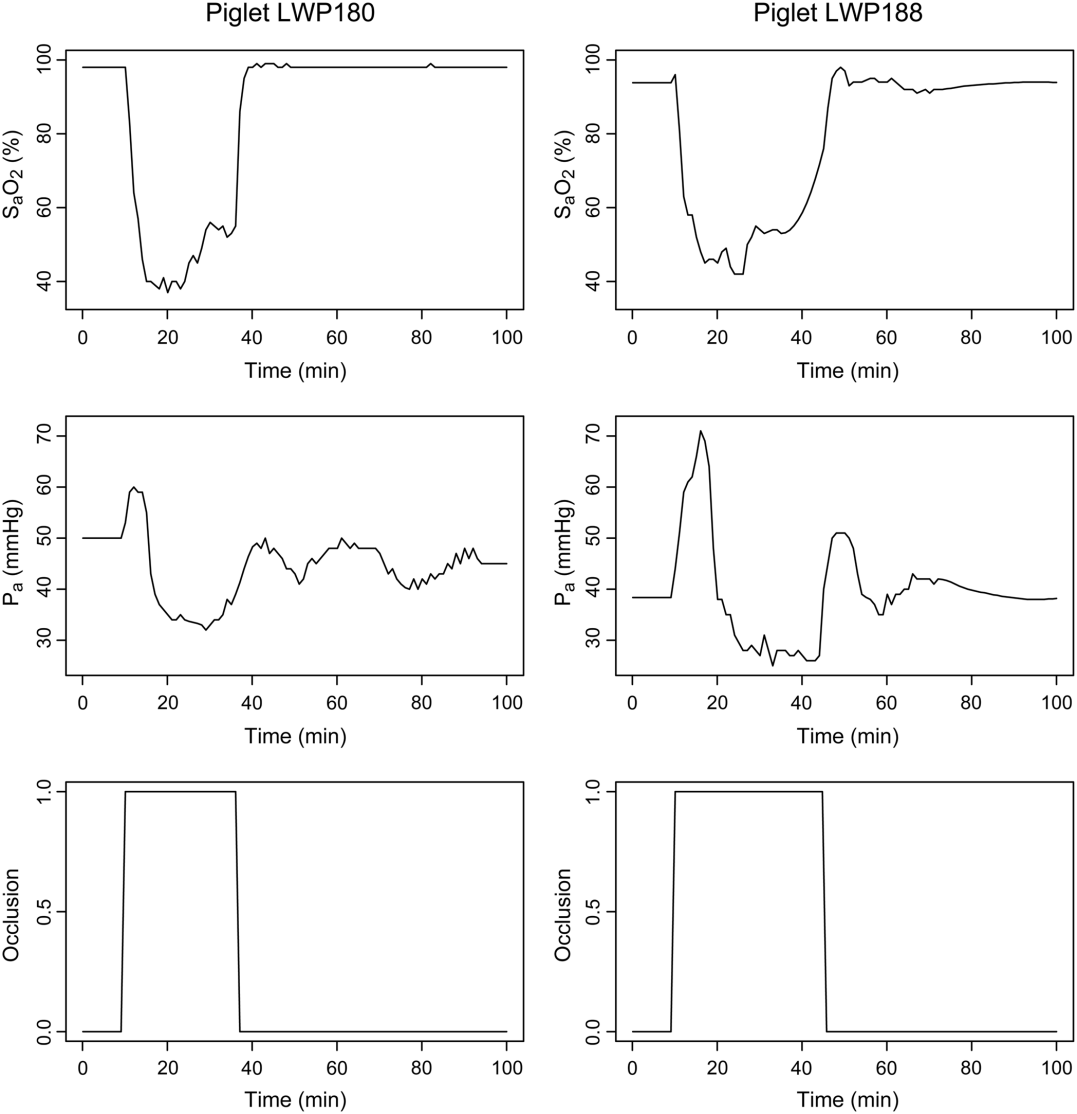
Inputs to the model for two example piglets. Time courses of measured arterial oxygen saturation (S_a_O_2_) and arterial blood pressure (P_a_), together with the degree of carotid artery occlusion, for piglets LWP180 (left) and LWP188 (right).

The measured SaO_2_ and P_a_ were used as inputs to the model. Arterial occlusion was represented by changing the control parameter k_occ_ from 0 to 1 over a minute and then decreasing it in the same way at the end of occlusion. Model outputs were compared with the results measured by NIRS and MRS. Simulations were initially carried out using the BRAINCIRC modelling environment (http://braincirc.sourceforge.net/), and more recently with its successor system, the Brain/Circulation Model Developer (http://tinyurl.com/ucl-bcmd). Model definitions and inputs are online at http://tinyurl.com/brainpiglet. Results from two piglets are presented as case studies, denoted LWP180, which showed good recovery following HI, and LWP188, which did not. The model inputs for both piglets are shown in Fig 4.

Sensitivity analysis was carried out on the signals from each piglet. We used a form of the Morris elementary effects method [46], a global sensitivity sampling scheme commonly used as a screening tool for large models because of its comparatively low cost in model evaluations. The variant employed was devised by Saltelli et al. [32] and is implemented using the R sensitivity package [47]. Briefly, the parameter space, with ranges specified for each parameter, is subdivided into a grid with the same number of divisions on each axis. A number of ‘trajectories’ are then navigated through this space, starting from a random grid position and taking a step along each dimension in random order until all parameters have been changed once. The model is evaluated at each step of the trajectory, and the sensitivity to each parameter estimated from model output changes associated with changes to that parameter across all sampled trajectories. Two statistics are notable: the mean of the absolute values of the changes, denoted *μ* ^⋆^, and their standard deviation, *σ*. The larger the value of *μ* ^⋆^ the more influential a parameter is on the output, while large *σ* indicates a non-linear influence of the parameter.

We wished to identify parameters to optimise for an improved fit of the model to the data. We therefore chose to use as the output the root mean square (RMS) difference between each measured signal and its modelled equivalent. The resulting *μ* ^⋆^ values were normalised for each signal by dividing by the maximum value.

Parameter ranges were chosen by using a default of ±20% of the normal model value. A larger range was chosen for parameters which were set heuristically and have a large uncertainty in their value. Some parameter ranges were also adjusted to keep their values within a meaningful range—for example, several parameters are defined to lie between 0 and 1. Finally, a few parameters were excluded from the analysis completely, either because they represent physical constants and therefore are known sufficiently accurately, or because they were not relevant to the current studies. The ranges used for each parameter can be found in the supplementary material. In total, 99 parameters were included, with 2000 repeats, giving a total of 200000 simulations. Simulations which failed to give a result were excluded from the analysis. Parameters with a normalised *μ* ^⋆^ of 0.5 or greater (averaged across both piglets) were then optimised.

Optimisation was carried out using the PSwarm method [48] to minimise the RMS difference between the modelled and measured signals on which the parameter had a strong influence. The parameters were optimised in groups depending on which signals they influenced, in order to reduce the dimensionality of the optimisations. Parameter values were limited to the same ranges used in the sensitivity analysis.

## Results

**Fig 5:**
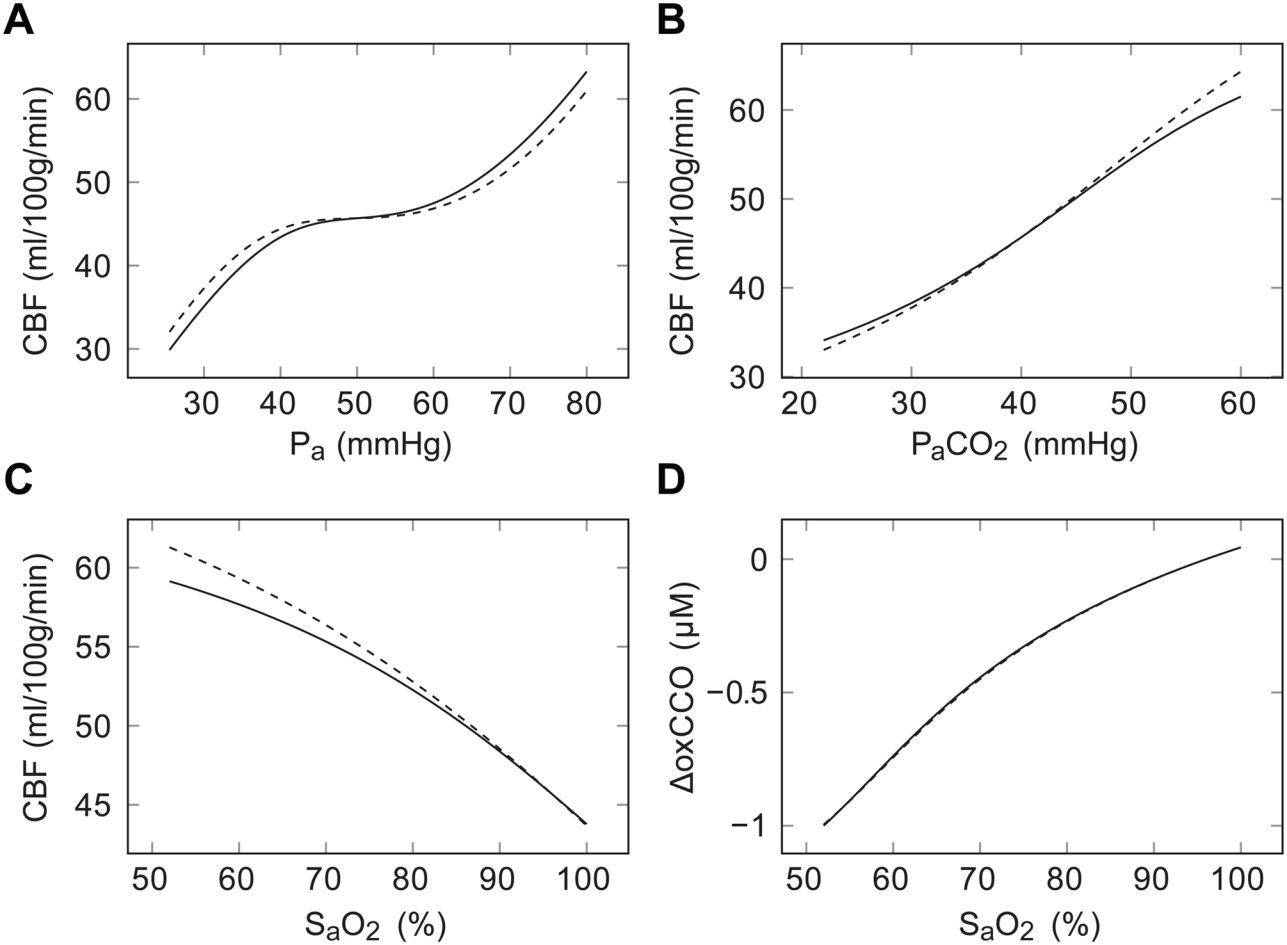
Steady state simulations before and after the model alterations. Plots show variation of (A) cerebral blood flow (CBF) with mean arterial pressure (P_a_); (B) CBF with partial pressure of carbon dioxide (P_a_CO_2_); CBF with arterial oxygen saturation (S_a_O_2_); and (D) oxidised cytochrome c oxidase (ΔoxCCO) with S_a_O_2_. In each case, the specified input parameter was varied while other conditions were held constant. Results from the earlier BrainPiglet model [31] are shown as dashed lines, while solid lines show those from the new version, BrainPigletHI.

The results from steady state simulations on the model are shown in Fig 5, together with equivalent results from the original model. The addition of a new arterial compartment gives rise to small differences in CBF response, but these are well within the range of experimental variability.

**Fig 6:**
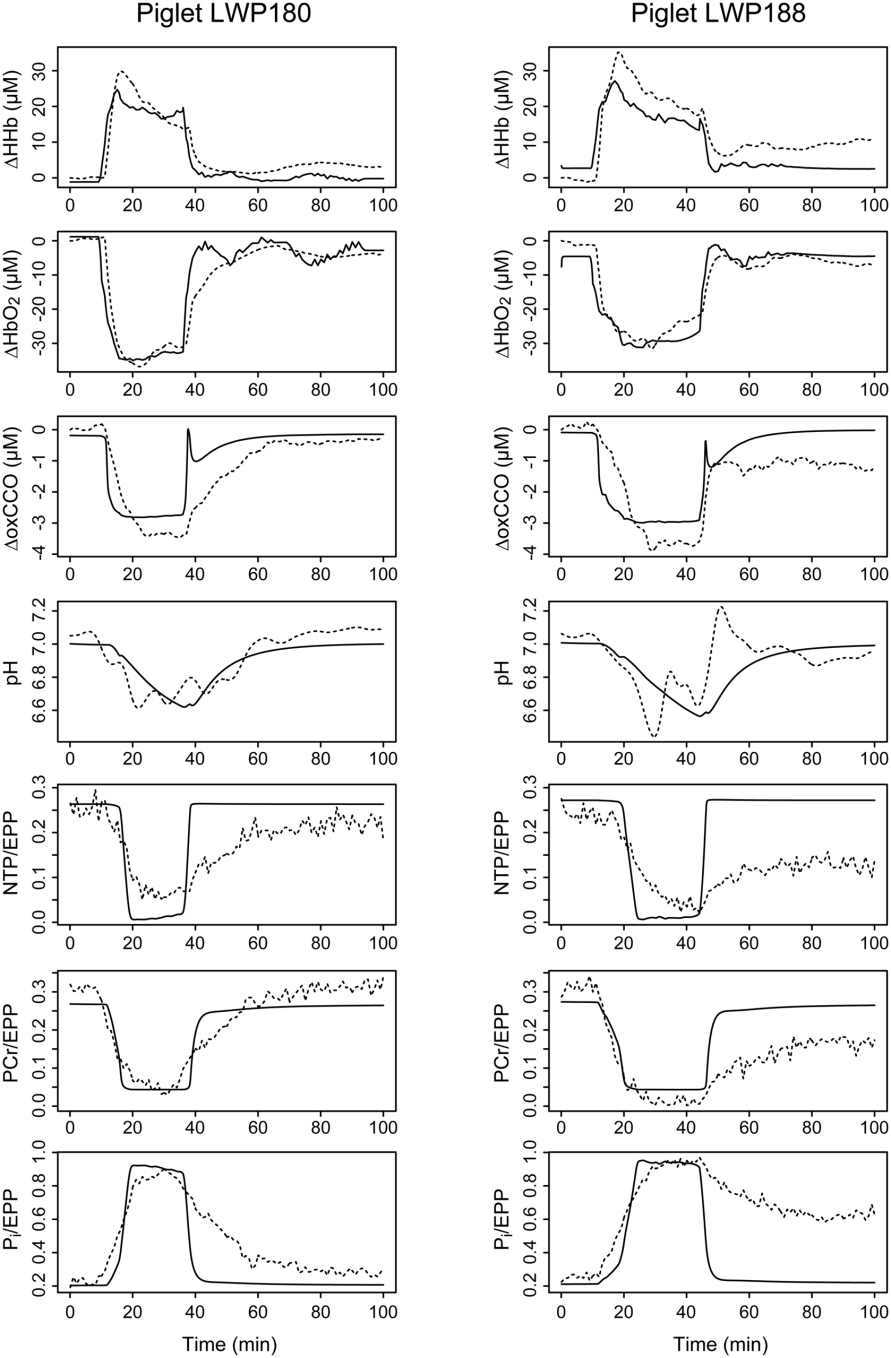
Comparison of optimised signals to experimental measurements for both piglets. Measured time courses are shown as dashed lines, and corresponding model outputs as solid lines, for the following signals (top to bottom): changes in deoxygenated haemoglobin (ΔHHb), oxygenated haemoglobin (ΔHbO2) and oxidised cytochrome c oxidase (ΔoxCCO); cytoplasmic pH; and the fractions of the exchangeable phosphate pool present as nucleotide triphosphate (NTP/EPP), phosphocreatine (PCr/EPP) and inorganic phosphate (P_i_/EPP). Results for the recovering piglet LWP180 are shown in the left column, while the right column shows those for the non-recovering piglet LWP188.

The experimentally measured signals from the two piglets can be seen as dashed lines in Fig 6. Both piglets showed a drop in ΔHbO_2_ and a rise in ΔHHb which both return close to their baseline values after the insult. Both piglets also show a reduction of CCO (a drop in ΔoxCCO) as expected. There is also a decrease in NTP/EPP and PCr/EPP and an increase in P_i_/EPP. These signals return to baseline for piglet LWP180 but not for piglet LWP188, indicating that this piglet did not recover.

Table 2 shows results from the sensitivity analysis for those parameters with a normalised *μ* ^⋆^ > 0.5 for the measured output signals. In all cases, as is common for Systems Biology models [34], sensitivity was dominated by a small number of parameters. Given their impact on the outputs, these parameters were assumed to capture significant aspects of the model behaviour and therefore be potentially informative of the physiological state. We return to this assumption in the Discussion.

**Table 2:** Main parameter sensitivities of the modelled signals.

	ΔHbO_2_	ΔHHb	CCO	NTP/EPP	PCr/EPP	P_i_/EPP	pH
[Hbtot]_n_	1.00	1.00					
r_0_	0.81						
V_blood,n_	0.61						
Cu_A,frac,n_			1.00				
[CCO]_tis_			0.93				
[ATP]_n_				1.00	1.00	0.67	
[PCr]_n_/[P_i_]_n_				0.75		1.00	
[PCr]_n_				0.83	0.56		0.66
pH_o,n_							1.00

Normalised *μ* ^⋆^ values greater than 0.5 from a sensitivity analysis of the model parameters. Output signals are shown at the top and model parameters to the left. A full description of all model parameters and the equations in which they occur can be found in S1 Text.

Based on the sensitivity results, the following optimisation strategy was chosen:

- Parameters [Hbtot]_n_, r_0_ and V_blood,n_ were optimised for the output signals ΔHbO_2_ and ΔHHb.
- Parameter pH_o,n_ was optimised for the cytoplasmic pH.
- Parameters [CCO]_tis_ and Cu_A,frac,n_ were optimised for the output signal ΔoxCCO.
- Parameters [ATP]_n_, [PCr]_n_ and [PCr]_n_/[P_i_]_n_ were optimised for the phosphate signals NTP/EPP, PCr/EPP and P_i_/EPP.

As noted previously, the measured CCO and ^31^P-MRS signals did not return to baseline following HI in piglet LWP188, implying a state change during the insult relative to piglet LWP180. For the initial optimisation we wished to consider only the model state prior to this change, so the model was optimised to these signals only up to the nadir of the insult.

**Fig 7:**
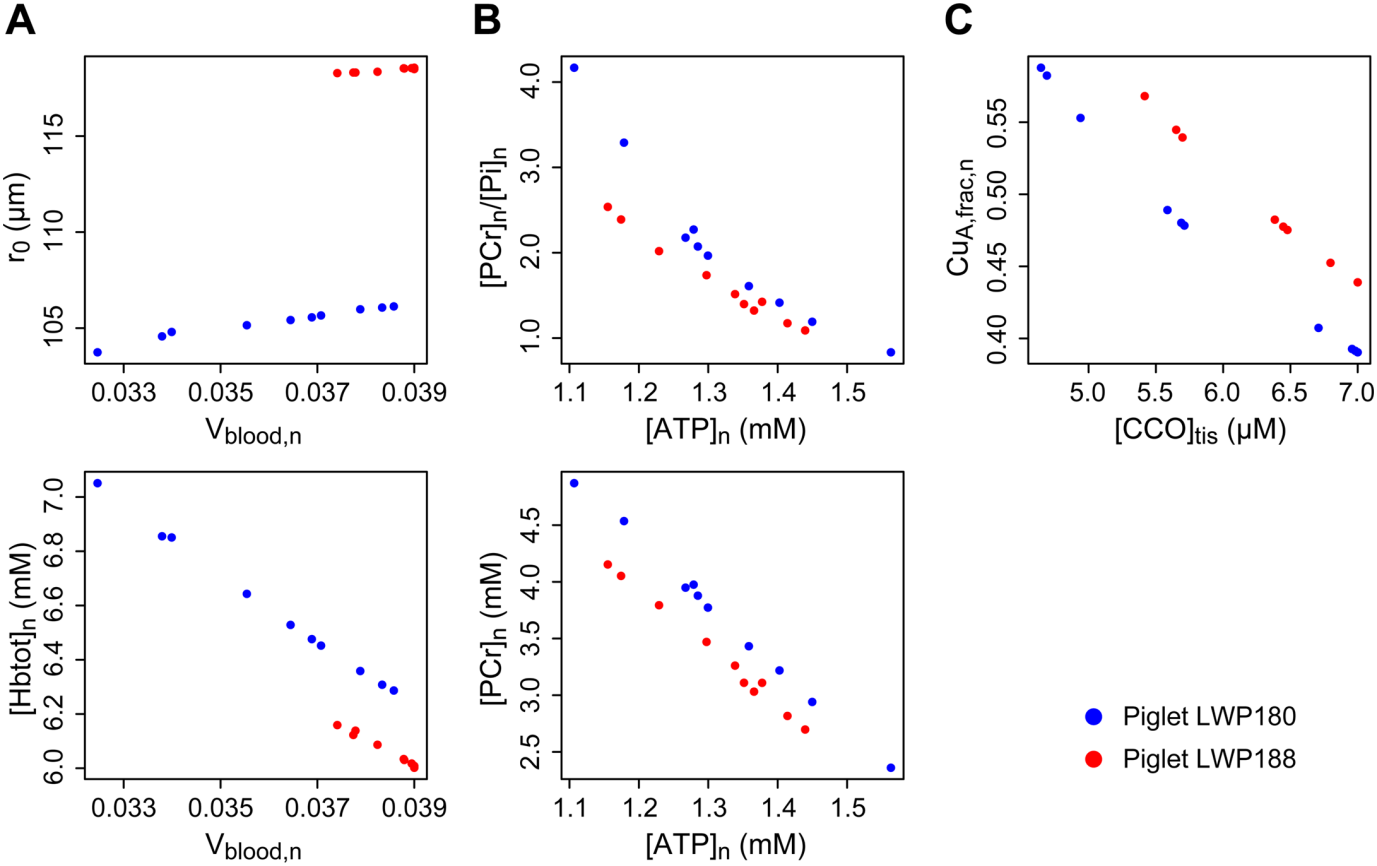
Correlations between optimised parameter values. Parameter estimates are shown from repeated optimisations to the measured signals: (A) ΔHbO2 and ΔHHb; (B) NTP/EPP, PCr/EPP and P_i_/EPP; (C) ΔoxCCO. Estimates exhibit significant variation and are also highly correlated, so results cannot be considered reliable estimators of the individual physical parameters. However, the values for each piglet occupy different regions of the parameter space, suggesting that they jointly identify distinct locations in a lower-dimensional space of behavioural variation.

Global optimisation of non-convex functions is not guaranteed to produce uniquely correct results [49]. Even when optimisation is apparently successful the parameters may suffer from problems of identifiability [50]. To assess the validity of the parameter estimates in this case, multiple optimisations were run with differing initial populations. The results are shown graphically in Fig 7, and summarised in Table 3. It can be seen that the optimised values are not unique, exhibiting significant variation in some instances, and are also not independent. We therefore cannot consider them good estimates of the true physical quantities. Nevertheless, the population results for the two piglets are visibly distinct, implying that the parameter ensemble jointly manifests a genuine difference of location in some other, lower-dimensional, space of behavioural variation [34, 35]. Characterisation of such spaces is an active area of modelling research, beyond the scope of the current work. Here, we simply accept the ensemble estimates as a useful proxy for that location.

**Table 3:** Parameter values before and after optimisation.

Parameter	Description	Default	LWP180 (mean ± SD)	LWP188 (mean ± SD)
[CCO]_tis_	Concentration of cytochrome c oxidase in tissue	2.2 μM	5.9 ± 1.0	6.4 ± 0.6
Cu_A,frac,n_	Normal oxidised fraction of Cu_A_ in cytochrome c oxidase	0.67	0.48 ± 0.08	0.49 ± 0.05
[Hbtot]_n_	Normal total haemoglobin concentration in blood	5.4 mM	6.6 ± 0.3	6.1 ± 0.1
r_0_	A radius in the elastic tension relationship	126 μm	105 ± 1	118 ± 0
V_blood,n_	Normal blood volume as a fraction of brain tissue	0.0325	0.036 ± 0.002	0.038 ± 0.001
[ATP]_n_	Normal concentration of ATP in the cytoplasm	2.2 mM	1.3 ± 0.1	1.3 ± 0.1
[PCr]_n_	Normal concentration of PCr in cytoplasm	2.6 mM	3.7 ± 0.7	3.3 ± 0.5
[PCr]_n_/[P_i_]_n_	Ratio of normal concentrations of PCr and P_i_ in the cytoplasm	2.73	2.1 ± 1.0	1.7 ± 0.5
pH_o,n_	Normal cytoplasmic pH	7.00	7.01 ± 0.0	7.03 ±0.0

Simulations using the optimised parameters for both piglets are shown as solid lines in Fig 6. For most of the signals the magnitude of the measured change is matched well by the model. For ΔoxCCO and the ^31^P-MRS measured variables, the modelled signals show changes occurring faster than is seen in the measurements. The modelled ΔoxCCO shows a smaller drop than seen in the measurement, and also an overshoot at the end of the occlusion, before the system equilibrates to a a more realistic value. These faster time courses may be a consequence of the model’s compartmental structure, which neglects spatial factors that could be expected to lead to a slower response propagation. Conversely, the pH measurements calculated from the ^31^P-MRS spectra appear to exhibit a faster and less stable response than the model prediction.

With no state change represented during HI, all modelled signals return to baseline once the insult ends. This is clearly not the case for the measured ΔoxCCO and ^31^P-MRS signals from the non-recovering piglet LWP188. To attempt to model this non-recovery, three possible changes were considered:

- non-recovery of some fraction of the occluded blood flow, represented by k_occ_
- a change in the rate of oxygen metabolism, mediated via mitochondrial uncoupling, represented by k_unc_
- the death of some proportion of the cells in the tissue compartment, represented by the parameter d_f_

For each scenario, the post-insult value of the relevant parameter was optimised jointly with the other parameters for the ΔoxCCO and ^31^P-MRS variables. The other parameters were assumed to remain the same after the insult as before, but optimisation was performed against the whole time course of the signal. Thus, the estimates take into account evidence from the response to the insult under the candidate scenario. In consequence, the predictions of the pre-insult behaviour can differ in each case, even though all three models would be the same as the original in the absence of HI.

**Fig 8:**
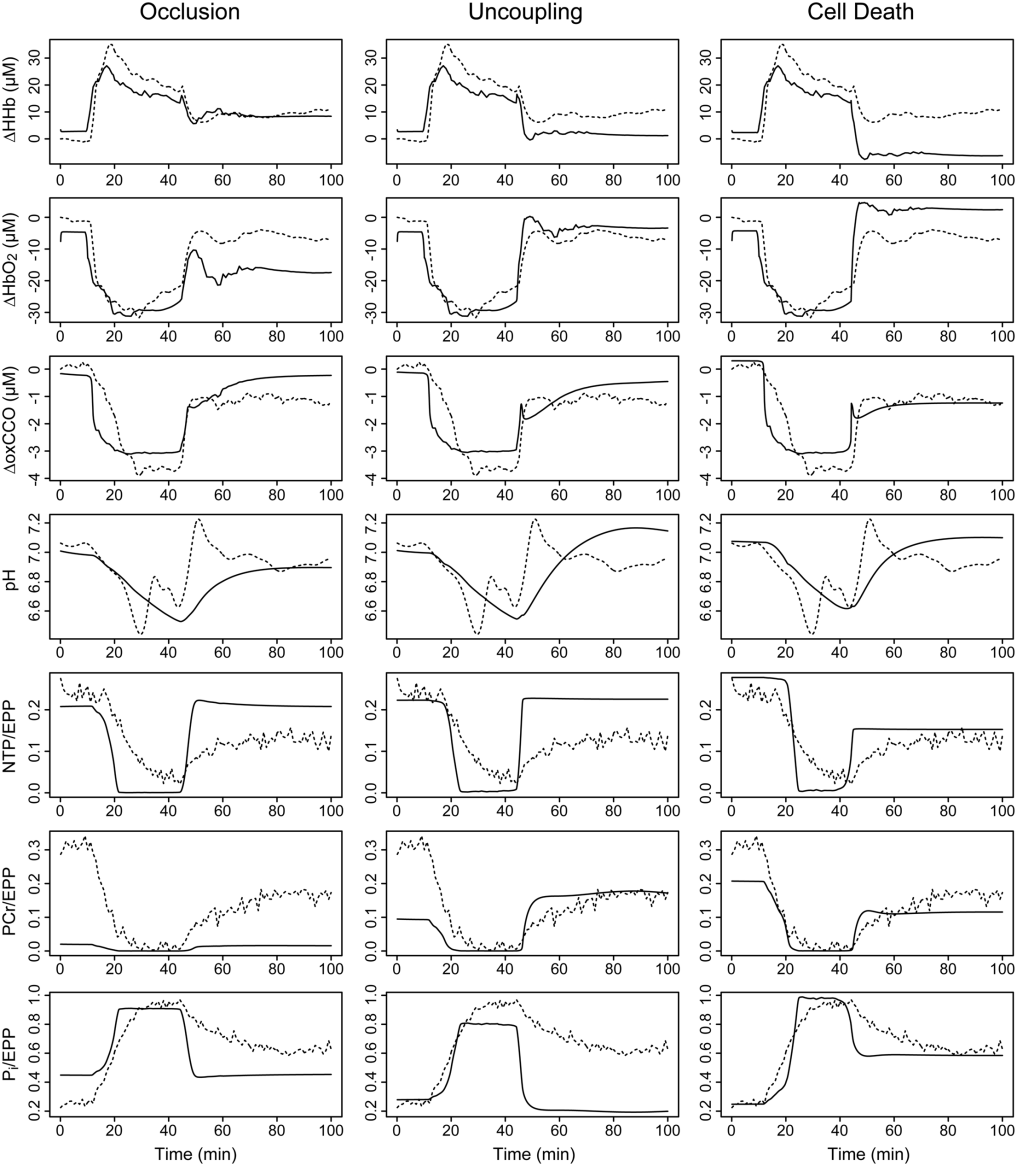
Modelled signals using a single non-recovery factor. Parameters affecting the ^31^P-MRS and ΔoxCCO signals were jointly optimised with a control parameter representing either (left) the post-insult level of arterial occlusion; (centre) mitochondrial uncoupling; or (right) cell death. Measured time courses are shown as dashed lines, and corresponding model outputs as solid lines, for the following signals (top to bottom): changes in deoxygenated haemoglobin (ΔHHb), oxygenated haemoglobin (ΔHbO2) and oxidised cytochrome c oxidase (ΔoxCCO); cytoplasmic pH; and the fractions of the exchangeable phosphate pool present as nucleotide triphosphate (NTP/EPP), phosphocreatine (PCr/EPP) and inorganic phosphate (P_i_/EPP).

**Fig 9:**
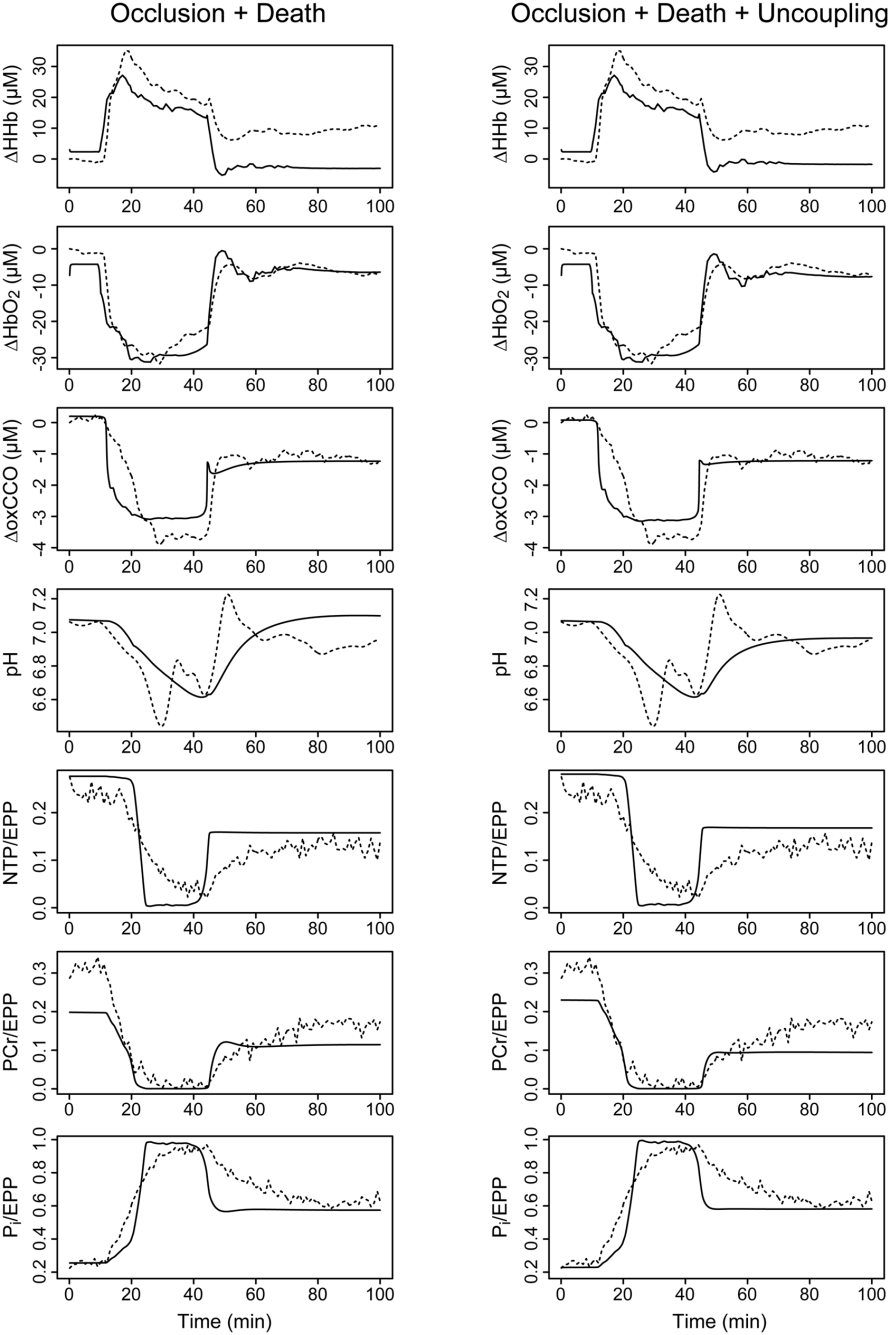
Modelled signals using combined non-recovery factors. Parameters affecting the ^31^P-MRS and ΔoxCCO signals were jointly optimised with control parameters for both cell death and arterial occlusion (left); and for cell death and occlusion combined with mitochondrial uncoupling (right). Measured time courses are shown as dashed lines, and corresponding model outputs as solid lines, for the following signals (top to bottom): changes in deoxygenated haemoglobin (ΔHHb), oxygenated haemoglobin (ΔHbO2) and oxidised cytochrome c oxidase (ΔoxCCO); cytoplasmic pH; and the fractions of the exchangeable phosphate pool present as nucleotide triphosphate (NTP/EPP), phosphocreatine (PCr/EPP) and inorganic phosphate (P_i_/EPP).

Optimisation results are given in Table 4. Corresponding model simulations for the single scenarios are shown in Fig 8, while Fig 9 shows model outputs for a scenario including both occlusion and cell death, and another including all three factors. As before, we cannot infer that the optimised parameters are physically correct, but it is instructive to observe the behavioural changes obtained in each scenario.

**Table 4:** Optimised parameters for LWP188 non-recovery scenarios.

Parameter	Occlusion	Uncoupling	Cell Death	Occl. & Death	All
[CCO]_tis_	0.7	0.7	5.9	4.9	3.9
Cu_A,frac,n_	0.46	0.45	0.54	0.65	0.83
[ATP]_n_	0.1	0.5	0.7	0.7	0.9
[PCr]_n_	0.8	4.7	5.8	5.9	5.5
[PCr]_n_/[P_i_]_n_	0.1	1.2	10	10	10
k_occ_	1	–	–	1	1
k_unc_	–	0.33	–	–	1.63
d_f_	–	–	0.45	0.43	0.40

Occlusion and uncoupling are individually able to produce only marginal changes, trading a slightly better fit in one signal for significant worsening in another. This is particularly evident in the phosphate signals, where trivial improvements in the post-insult NTP and P_i_ behaviour are achieved at the cost of very poor simulation of PCr (Fig 8, bottom three rows). Neither scenario reproduces the failure of the CCO and phosphate signals to return to baseline following the insult. The cell death model, on the other hand, is much better able to reproduce the failure to return to baseline in these outputs, but leads to an over-recovery of the haemoglobin signals, probably due to the reduced oxygen consumption, and somewhat overestimates the cytoplasmic pH. These failings are partly compensated when the state changes are combined, although this may simply be a consequence of having more parameters with which to fit.

While the accuracy of the optimised parameter values is unknown, it is worth noting that in every optimisation including k_occ_, the optimised value indicates that the arteries remain occluded.

## Discussion

BrainPigletHI is a significant expansion of our earlier model of circulation and metabolism in the piglet brain, BrainPiglet [31]. Like its predecessor, the model is intended to provide a computational tool that can simulate our instrumental measurements in piglets, in this case during a hypoxic-ischaemic insult. It allows integration of multimodal measurements and more in depth investigation of the physiological and biochemical changes that occur during this challenge. We note this focus is much narrower than that of developing an animal preclinical experimental model of *human* neonatal hypoxic-ischaemia, a very active field of research that extends both in the type of animals used (rats, mice, fetal sheep)[51] but also the type of experimental challenge (e.g. hypoxia versus hypoxia and carotid occlusion). [52] Such development is beyond the current and future scope of our work.

New features have been added to BrainPiglet to allow the modelling of experimentally-induced HI and improve the simulation of multimodal data from broadband NIRS and ^31^P-MRS, including cytoplasmic pH. The model outputs have been compared with measurements from piglet studies in which different outcomes occurred after HI, and used to investigate possible mechanisms for the observed behaviour.

For the first time in this type of modelling, we have used sensitivity analysis to identify parameters with a strong effect on the model’s ability to reproduce experimental results. These parameters were then optimised to characterise the differences between the piglets. Implicitly, we are assuming that the model structure represents the fundamental similarities of behaviour shared across all individuals, while a small number of influential parameters can capture interesting behavioural variations between them.

We note that the Morris sensitivity analysis is a sampling-based method that provides only an estimate of the parameter sensitivities. It is widely used because of its relative efficiency in terms of number of simulations required. Results may vary for different sample runs, especially for complex models with highly non-uniform parameter spaces. The outcome is also dependent on the parameter ranges chosen. This is true for most sensitivity analysis methods: if the range of a parameter is expanded, then it will be adjusted by greater increments and its apparent influence is likely to increase. For ‘sloppy’ models like BrainPigletHI, with strongly skewed sensitivity distributions [34], this issue should not be severe, since most parameters will remain of low importance over any physiologically-plausible range. However, the set of influential parameters identified by our analysis is not exhaustive. In (smaller) tests with altered ranges, the result set was overlapping but non-identical. The set chosen appears to capture enough variability to be useful, but we cannot rule out the possibility that some important dimension of behaviour has been excluded. A complete determination of parameter influences, and their relation to the true dimensions of behavioural variability in the model, is well beyond the scope of this paper—if it were possible at all.

Most of the influential parameters in the chosen set represent normal concentrations of metabolites. This is consistent with our expectations of the model behaviour. These are also values that might be expected to vary between individuals, making them appropriate parameters to change when optimising the model to datasets from different subjects.

It may reasonably be argued that parameter fitting to the individual datasets presents a problem of validation, since there is no independent data against which the fit may be compared. In the context of single experiments, with a single intervention after which the subject is sacrificed, there is limited scope for using a classic ‘training data’/‘test data’ paradigm. In the longer term, and in particularly for applications to human subjects, we would expect to employ an initial training phase, in which the model is parameterised for the individual, and subsequent application phases, where the individualised model is used for prediction. Even in such a scheme, however, the dynamic variability of physiological systems is such that training is unlikely ever to be a one-off process, instead requiring constant monitoring and updating.

Uncertainty in parameter estimation may arise from many different sources: measurement errors in the data, specification errors in the model design, structural non-identifiability of interacting parameters, non-convergence of the optimisation procedure. Considering only the latter concerns, it is evident from the results in Fig 7 that the true physical values corresponding to the parameters cannot by determined with any confidence from our model fitting. Nevertheless, the parameter ensemble can usefully distinguish behavioural differences. Once again, this is consistent with the known properties of ‘sloppy’ models [34].

With an optimised set of influential parameters, BrainPigletHI was able to approximately reproduce the measured responses of piglet LWP180, which recovered after the HI insult, although there were differences of time course. In particular, the model’s responses were typically faster than those seen in the real data, particularly in the release phase. This may be due to the lack of spatial representation in the model. The different model compartments are considered purely as functional units, responding all in one go, rather than as the spatially-distributed systems they actually are. This omission may be addressed in a future model version, but it is likely to require a fundamental change in the model structure. For the moment, we choose to accept the difference in response times as a reasonable trade-off against significantly increased model complexity.

For the non-recovering piglet LWP188, the model was able to reproduce the recorded behaviour up to the end of the insult, but the subsequent failure of the ^31^P-MRS and ΔoxCCO signals to return to baseline required the assumption that an internal state change occurred during HI. We investigated three candidate changes to the model state, corresponding to possible physiological explanations for the failure to recover: disrupted blood flow; altered oxygen consumption; the death of a proportion of the cells. Neither blood flow nor mitochondrial uncoupling were able to simulate the non-recovery, suggesting that cell death was indeed a factor.

Indeed if the hypothesis of cell death is correct, there are two likely explanations for the observed results. Firstly, microvascular shunting could occur, causing blood to no longer flow to all parts of the brain. This would cause a decrease in CBF and therefore a decrease in oxygen delivery, despite normal S_a_O_2_ levels. Secondly, CMRO_2_ in the remaining functioning cells may be greatly increased. This could occur as a result of uncoupling between the reduction of oxygen and the synthesis of ATP in the mitochondria. Uncoupling is known to occur after hypoxic or ischaemic injury [53]. This would normally be expected to increase oxidation of CCO. However, if uncoupling occurred alongside cell death, the oxidation could be masked by the larger reduction effect.

If the assumption of cell death is incorrect, it is more difficult to explain the relationship between ΔoxCCO and NTP/EPP measurements. Uncoupling in the mitochondria could lead to a reduced NTP concentration and a small oxidation of CuA; that has also been observed experimentally after the administration of an uncoupler (dinitrophenol) to newborn piglets [54]. Other possible explanations for the results include an impairment in glycolysis or the TCA cycle, but this would lead to an oxidation in CCO. A large decrease in oxygen delivery relative to baseline may also explain the results. CBF measurements would help to resolve this question.

The simulations with cell death did not, of course, exactly reproduce the measured signals, and the optimisation results do not constitute proof. The models of the state changes in all three cases were somewhat simplistic. It is likely that the observed behaviour did not result exclusively from a single isolated cause. We note that in all optimisations where blood flow disruption was permitted, the results indicated that it did indeed occur. The best simulations were those in which all three factors played a part, although this may simply be a result of having more opportunities to overfit. Ultimately, this is not a question that can be settled by modelling alone. The model can only suggest mechanisms that merit future experimental investigation.

Extending the model to simulate cytoplasmic pH has produced promising initial results. We assume that pH_Pi–PEt_ is a reasonable comparison. Strictly, ^31^P-MRS measurements represent an average over all the tissue compartments in which the metabolites are found. However, the cytoplasm has a much larger volume than the mitochondria, and is therefore expected to contribute more to the measurement. Our modelled pH and the measured pH_Pi–PEt_ show comparable drops during the insult for both piglets, though the model is slower to respond. A recent model of intracellular pH by Orlowski et al. showed a similar drop in pH following a reduction in CBF [25]. That model investigated the effect of ischaemia in neurons and astrocytes and incorporated the dynamics of carbonic acid, the sodium-potassium pump and the sodium hydrogen exchanger (NHE). NHE is known to play an important role during hypoxia-ischaemia—animal models have shown NHE inhibitors to be neuroprotective [55]. We are currently working on improving the simulation of intracellular pH in our model by including these components and processes and by comparing the model with measurements from more piglets.

Several factors need to be borne in mind when considering the data analysed here. Firstly, the NIRS and MRS measurements do not come from exactly the same area of the brain and it is known that HI damages different parts of the brain to different extents. Secondly, there are other changes to the brain following HI. In particular, cerebral oedema is likely to occur [56] which could have an effect on the measurements. Finally, the haemoglobin measurements may also be influenced by changes in haematocrit that have taken place during or after the insult. The simple method used to simulate cell death here is not capable of simulating the pH changes that would occur as a consequence of cell death. Future model developments will explore these in more detail, allowing for simulation of distinct cell populations with different fates.

Other model improvements are currently under investigation. We have used total cytosolic NAD and NADH concentrations in the modelling of intracellular pH. It has recently come to our attention that the free unbound cytosolic NAD and NADH concentrations may be more more relevant. Sensitivity analysis indicates that these values do not have a large impact on the results, but a future version of the model will be altered to use to more correct values. The scope of the model will also be extended further to allow simulation of secondary energy failure, the period during which much of the long-term brain damage caused by HI is thought to occur. This will allow the model to be used to investigate treatments of HI such as hypothermia and to simulate the effects of specific drugs such as melatonin [57]. Ultimately we aim to adapt the model to human infant physiology and employ it to aid the interpretation of multimodal measurements in neonatal HI.

## Supporting Information

S1 Text BrainPigletHI model definition. Full list of the model equations and parameter values.
(PDF)
