# Supplementary material for: Modelling Blood Flow and Metabolism in the Preclinical Neonatal Brain during and Following Hypoxic-Ischaemia
Source: PLoS One. 2015 Oct 7;10(10):e0140171. doi: 10.1371/journal.pone.0140171 (PMC4596480; doi:10.1371/journal.pone.0140171)
Supplement: S1 Text — Full list of the model equations and parameter values. (PDF) [file pone.0140171.s001.pdf]

Supplementary material

1 Graph diagram of model

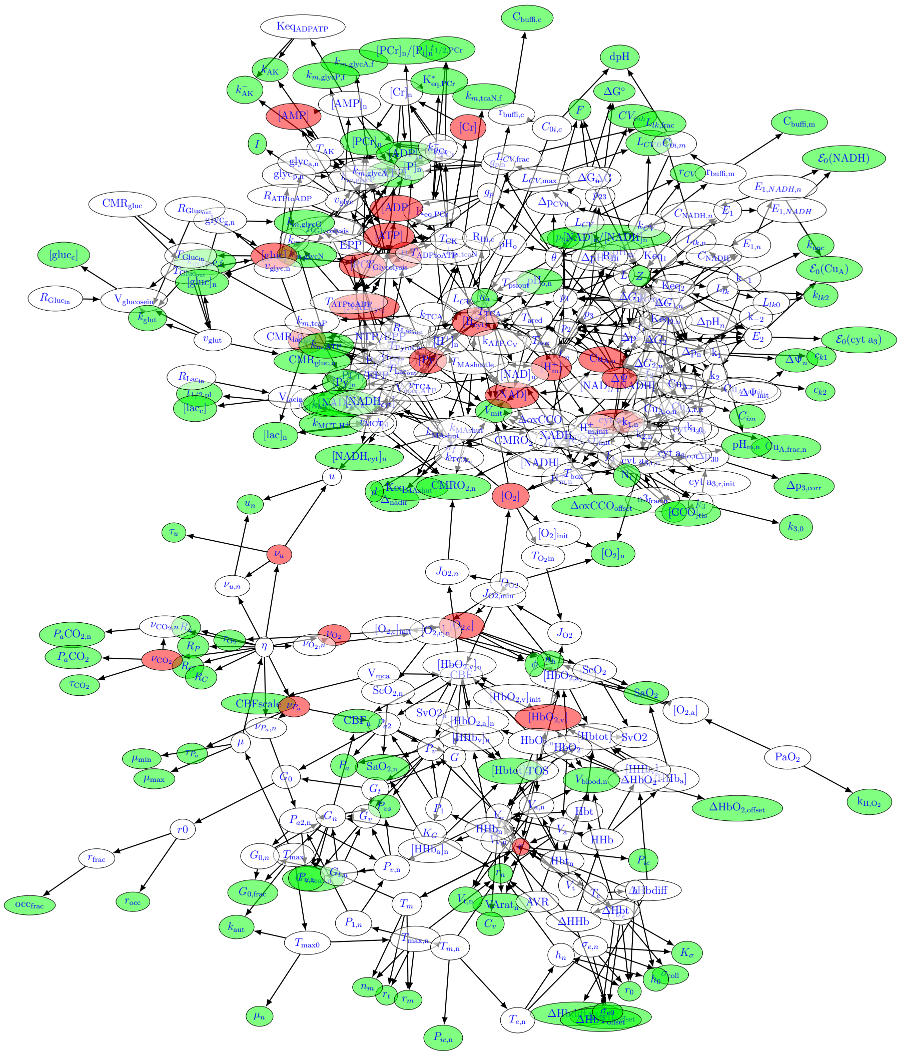

Figure 1: A graph showing of the model showing the connections between the parameters (white) variables (red) and temporary variables (green)

2 Pressure profile along the arteries and veins

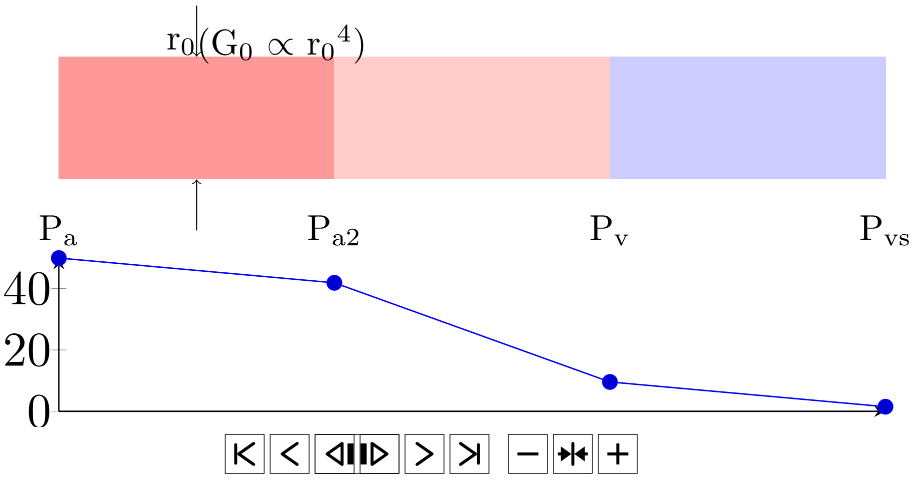

Figure 2: An illustration of how the blood pressure changes through the different compartments and the relative changes in the volumes of the supplying arterial, arterial and venous compartments. Animation (available in some pdf readers) shows how these change as the extent of artery occlusion is varied. Occlusion of the carotid arteries is equivalent to r_0 of approximately 1/3 of its baseline value.

3 BrainPiglet parameters and Variables

Contents

3.1 Differential Equations 2
3.2 Chemical Reactions 4
3.3 Variables 6
3.4 Temporary Variables 7
3.5 Parameters 16
3.6 Derived Parameters 21

3.1 Differential Equations

$$\frac{\mathrm{d}\mathrm{Cu}_{\mathrm{A,o}}}{\mathrm{d}t} = 4T_{\mathrm{aox}} - 4T_{\mathrm{ared}} \tag{1}$$

$$\frac{\mathrm{d}[\mathrm{ADP}]}{\mathrm{d}t} = -T_{\mathrm{CK}} - T_{\mathrm{ADPtoATP}} + T_{\mathrm{ATPtoADP}} - 2T_{\mathrm{AK}} - 2T_{\mathrm{Glycolysis}} \tag{2}$$

$$\frac{\mathrm{d}[\mathrm{AMP}]}{\mathrm{d}t} = T_{\mathrm{AK}} \tag{3}$$

$$\frac{\mathrm{d}[\mathrm{ATP}]}{\mathrm{d}t} = T_{\mathrm{CK}} + T_{\mathrm{ADPtoATP}} - T_{\mathrm{ATPtoADP}} + T_{\mathrm{AK}} + 2T_{\mathrm{Glycolysis}} \tag{4}$$

$$\frac{\mathrm{d}\,\mathrm{cyt}\ \mathrm{a}_{3,\mathrm{r}}}{\mathrm{d}t} = 4T_{\mathrm{aox}} - 4T_{\mathrm{box}} \tag{5}$$

$$\frac{\mathrm{d}[\mathrm{Cr}]}{\mathrm{d}t} = T_{\mathrm{CK}} \tag{6}$$

$$\frac{\mathrm{d}\Delta\Psi}{\mathrm{d}t} = \frac{p_2 f_2 + p_1 f_1 + p_3 f_3 - L}{C_{im}} \tag{7}$$

$$\frac{\mathrm{d}[\mathrm{gluc}]}{\mathrm{d}t} = -T_{\mathrm{Glycolysis}} + T_{\mathrm{Gluc_{in}}} - T_{\mathrm{Gluc_{out}}} \tag{8}$$

$$\frac{\mathrm{d}[\mathrm{H}^+_{\mathrm{m}}]}{\mathrm{d}t} = \frac{-p_2 T_{\mathrm{aox}}}{\mathrm{R_{Hi}}} - \frac{\left(p_1 + \frac{10}{6}\right) T_{\mathrm{ared}}}{\mathrm{R_{Hi}}} - \frac{p_3 T_{\mathrm{box}}}{\mathrm{R_{Hi}}} + \frac{T_{\mathrm{psiout}}}{\mathrm{R_{Hi}}} + \frac{4T_{\mathrm{TCA}}}{\mathrm{R_{Hi}}} + \frac{T_{\mathrm{MAshuttle}}}{\mathrm{R_{Hi}} V_{\mathrm{mit}}} \tag{9}$$

$$\begin{aligned}\frac{\mathrm{d}[\mathrm{H}^+_{\mathrm{cyt}}]}{\mathrm{d}t} = {} & \frac{p_2 T_{\mathrm{aox}} V_{\mathrm{mit}}}{\mathrm{R_{Hi,c}}} + \frac{(p_1 + 4)\, T_{\mathrm{ared}} V_{\mathrm{mit}}}{\mathrm{R_{Hi,c}}} - \frac{T_{\mathrm{psiout}} V_{\mathrm{mit}}}{\mathrm{R_{Hi,c}}} - \frac{T_{\mathrm{CK}}}{\mathrm{R_{Hi,c}}} + \frac{4T_{\mathrm{Glycolysis}}}{\mathrm{R_{Hi,c}}} \\ & - \frac{T_{\mathrm{PytoLac}}}{\mathrm{R_{Hi,c}}} - \frac{T_{\mathrm{TCA}} V_{\mathrm{mit}}}{\mathrm{R_{Hi,c}}} + \frac{T_{\mathrm{Lac_{in}}}}{\mathrm{R_{Hi,c}}} - \frac{T_{\mathrm{Lac_{out}}}}{\mathrm{R_{Hi,c}}} - \frac{T_{\mathrm{MAshuttle}}}{\mathrm{R_{Hi,c}}}\end{aligned} \tag{10}$$

$$\frac{\mathrm{d}[\mathrm{lac}]}{\mathrm{d}t} = T_{\mathrm{PytoLac}} + T_{\mathrm{Lac_{in}}} - T_{\mathrm{Lac_{out}}} \tag{11}$$

$$\frac{\mathrm{d}[\mathrm{NAD}]}{\mathrm{d}t} = 2T_{\mathrm{ared}} - 5T_{\mathrm{TCA}} - \frac{T_{\mathrm{MAshuttle}}}{V_{\mathrm{mit}}} \tag{12}$$

$$\frac{\mathrm{d}[\mathrm{NAD_{cyt}}]}{\mathrm{d}t} = -2T_{\mathrm{Glycolysis}} + T_{\mathrm{PytoLac}} + T_{\mathrm{MAshuttle}} \tag{13}$$

$$\frac{\mathrm{d}[\mathrm{O_2}]}{\mathrm{d}t} = -T_{\mathrm{box}} + \frac{T_{\mathrm{O_2 in}}}{V_{\mathrm{mit}}\,(1 - d_f)} \tag{14}$$

$$\frac{\mathrm{d}[\mathrm{P_i}]}{\mathrm{d}t} = -T_{\mathrm{ADPtoATP}} + T_{\mathrm{ATPtoADP}} - 2T_{\mathrm{Glycolysis}} \tag{15}$$

$$\frac{\mathrm{d}[\mathrm{PCr}]}{\mathrm{d}t} = -T_{\mathrm{CK}} \tag{16}$$

$$\frac{\mathrm{d}[\mathrm{Py}]}{\mathrm{d}t} = 2T_{\mathrm{Glycolysis}} - T_{\mathrm{PytoLac}} - \frac{T_{\mathrm{TCA}} V_{\mathrm{mit}}}{1} \tag{17}$$

$$\frac{\mathrm{d}\nu_{\mathrm{CO_2}}}{\mathrm{d}t} = \frac{1}{\tau_{\mathrm{CO_2}}}\left(P_a\mathrm{CO_2} - \nu_{\mathrm{CO_2}}\right) \tag{18}$$

$$\frac{\mathrm{d}\nu_{\mathrm{O_2}}}{\mathrm{d}t} = \frac{1}{\tau_{\mathrm{O_2}}}\left([\mathrm{O_{2,c}}] - \nu_{\mathrm{O_2}}\right) \tag{19}$$

$$\frac{\mathrm{d}\nu_{P_a}}{\mathrm{d}t} = \frac{1}{\tau_{P_a}}\left(P_{a2} - \nu_{P_a}\right) \tag{20}$$

$$\frac{\mathrm{d}\nu_u}{\mathrm{d}t} = \frac{1}{\tau_u}\left(u - \nu_u\right) \tag{21}$$

3.1.1 Algebraic Equations

$$\phi\left(\frac{\mathrm{ScO_2}}{1-\mathrm{ScO_2}}\right)^{1/n_h} - [\mathrm{O_{2,c}}] = 0 \tag{22}$$

$$T_e + T_m - (P_1 - P_{ic})\, r = 0 \tag{23}$$

$$\mathrm{CBF}\,\left([\mathrm{HbO_{2,a}}] - [\mathrm{HbO_{2,v}}]\right) - J_{\mathrm{O2}} = 0 \tag{24}$$

3.2 Chemical Reactions

Reactions are represented as terms in differential equations (see section 3.1).

$$\mathrm{AK} : 2\,\mathrm{ADP} \rightleftharpoons \mathrm{ATP} + \mathrm{AMP} \tag{25}$$

a two way reaction in which two molecules of ADP are converted into one of ATP and one of AMP catalysed by adenylate kinase (Rate T_{AK})

$$\mathrm{ADPtoATP} : \mathrm{ADP} + \mathrm{P_i} \rightarrow \mathrm{ATP} \tag{26}$$

phosphorylation of ADP by Complex V (Rate T_{ADPtoATP})

$$\mathrm{aox} : p_2\,\mathrm{H^+_m} \rightarrow 4\,\mathrm{Cu_{A,o}} + 4\,\mathrm{cyt\ a_{3,r}} + p_2\,\mathrm{H^+_{cyt}} \tag{27}$$

the reaction in which $\mathrm{cyt\ a_{3,o}}$ oxidises $\mathrm{Cu_{A,r}}$ and p_2 protons are pumped out of the matrix (Rate T_{aox})

$$\mathrm{ared} : 4\,\mathrm{Cu_{A,o}} + (p_1 + \frac{10}{6})\,\mathrm{H^+_m} \rightarrow (p_1 + 4)\,\mathrm{H^+_{cyt}} + 2\,\mathrm{NAD} \tag{28}$$

the reaction in which $\mathrm{Cu_{A,o}}$ is reduced and p_1 protons are pumped out of the matrix (Rate T_{ared})

$$\mathrm{ATPtoADP} : \mathrm{ATP} \rightarrow \mathrm{ADP} + \mathrm{P_i} \tag{29}$$

hydrolysis of ATP (Rate T_{ATPtoADP})

$$\mathrm{box} : p_3\,\mathrm{H^+_m} + 4\,\mathrm{cyt\ a_{3,r}} + \mathrm{O_2} \rightarrow \tag{30}$$

the reaction in which $\mathrm{O_2}$ oxidises $\mathrm{cyt\ a_{3,r}}$ and p_3 protons are pumped out of the matrix (Rate T_{box})

$$\mathrm{Gluc_{in}} : \rightarrow \mathrm{gluc} \tag{31}$$

transport of glucose from the blood to the cytoplasm (fixed rate) (Rate $T_{\mathrm{Gluc_{in}}}$)

$$\text{Gluc}_{\text{out}} : \text{gluc} \rightarrow \tag{32}$$

transport of glucose from the cytoplasm to the blood (Rate $T_{\text{Gluc}_{\text{out}}}$)

$$\text{Glycolysis} : \text{gluc} + 2\,\text{ADP} + 2\,\text{NAD}_{\text{cyt}} + 2\,\text{P}_{\text{i}} \rightarrow 2\,\text{ATP} + 4\,\text{H}^{+}_{\text{cyt}} + 2\,\text{Py} \tag{33}$$

a one way Michaelis Menten reaction which is assumed to capture the process of glycolysis (Rate $T_{\text{Glycolysis}}$)

$$\text{Lac}_{\text{in}} :\rightarrow \text{lac} + \text{H}^{+}_{\text{cyt}} \tag{34}$$

transport of lactate from the blood to the cytoplasm (fixed rate) (Rate $T_{\text{Lac}_{\text{in}}}$)

$$\text{Lac}_{\text{out}} : \text{lac} + \text{H}^{+}_{\text{cyt}} \rightarrow \tag{35}$$

transport of lactate from the cytoplasm to the blood (Rate $T_{\text{Lac}_{\text{out}}}$)

$$\text{MAshuttle} : \text{H}^{+}_{\text{cyt}} + \text{NAD} \rightleftharpoons \text{H}^{+}_{\text{m}} + \text{NAD}_{\text{cyt}} \tag{36}$$

malate aspartate shuttle: transfer between NAD/NADH in the cytoplasm and the mitochondria (Rate $T_{\text{MAshuttle}}$)

$$\text{O}_2\text{in} :\rightarrow \text{O}_2 \tag{37}$$

the reaction in which oxygen is supplied to the mitochondria (Rate $T_{\text{O}_2\text{in}}$)

$$\text{CK} : \text{PCr} + \text{ADP} + \text{H}^{+}_{\text{cyt}} \rightleftharpoons \text{Cr} + \text{ATP} \tag{38}$$

a two way mass action reaction representing the interconversion of PCr and ADP to Cr and ATP (Rate T_{CK})

$$\text{psiout} : \text{H}^{+}_{\text{cyt}} \rightarrow \text{H}^{+}_{\text{m}} \tag{39}$$

the reaction in which protons re-enter the matrix (via leak and Complex V) (Rate T_{psiout})

$$\text{PytoLac} : \text{H}^{+}_{\text{cyt}} + \text{Py} \rightleftharpoons \text{lac} + \text{NAD}_{\text{cyt}} \tag{40}$$

interconversion between pyruvate and lactate (Rate T_{PytoLac})

$$\text{TCA} : 5\,\text{NAD} + \text{Py} + \text{H}^{+}_{\text{cyt}} \rightarrow 4\,\text{H}^{+}_{\text{m}} \tag{41}$$

one step simple representation of pyruvate dehyrogenase and the TCA cycle (Rate T_{TCA})

3.3 Variables

$Cu_{A,o}$ (mM) Inital Value = $Cu_{A,o,init}$ = 0.022 mM
  the concentration of oxidised cytochrome-c-oxidase
[ADP] (mM) Inital Value = $[ADP]_n$ = 0.012 mM
  ADP concentration in cytoplasm
[AMP] (mM) Inital Value = $[AMP]_n$ = 0.000334 mM
  AMP concentration in cytoplasm
[ATP] (mM) Inital Value = $[ATP]_n$ = 1.2 mM
  ATP concentration in cytoplasm
cyt $a_{3,r}$ (mM) Inital Value = cyt $a_{3,r,init}$ = 0.000828 mM
  concentration of reduced cytochrome a_3 in mitochondria
[Cr] (mM) Inital Value = $[Cr]_n$ = 4.316 mM
  creatine concentration in cytoplasm
$\Delta\Psi$ (mV) Inital Value = $\Delta\Psi_{init}$ = 145 mV
  mitochondrial inner membrane potential
[gluc] (mM) Inital Value = $[gluc]_n$ = 1.2 mM
  concentration of glucose in the cytoplasm
$[H^+_m]$ (mM) Inital Value = $H^+_{m,init}$ = $3.981e-05$ mM
  concentration of hydrogen ions in mitochondria
$[H^+_{cyt}]$ (mM) Inital Value = $[H^+]_n$ = 0.0001 mM
  concentration of hydrogen ions in the cytoplasm
[lac] (mM) Inital Value = $[lac]_n$ = 3 mM
  concentration of lactate in the cytoplasm
[NAD] (mM) Inital Value = $[NAD]_n$ = 2.7 mM
  concentration of NAD in the mitochondria
$[NAD_{cyt}]$ (mM) Inital Value = $[NAD_{cyt}]_n$ = 359 mM
  concentration of NAD in the cytoplasm
$[O_2]$ (mM) Inital Value = $[O_2]_{init}$ = 0.024 mM
  the concentration of oxygen in the mitochondria
$[O_{2,c}]$ (mM) Inital Value = $[O_{2,c}]_{init}$ = 0.05343 mM
  the concentration of oxygen in the capillary
$[P_i]$ (mM) Inital Value = $[P]_n$ = 0.9524 mM
  inorganic phosphate concentration in cytoplasm
[PCr] (mM) Inital Value = $[PCr]_n$ = 2.6 mM
  phosphocreatine concentration in cytoplasm
[Py] (mM) Inital Value = $[Py]_n$ = 0.1 mM
  concentration of pyruvate in the cytoplasm
r (cm) Inital Value = r_n = 0.0187 cm
  typical radius of cerebral vessels
ν_{CO_2} (mmHg) Inital Value = $\nu_{CO_2,n}$ = 40 mmHg
  P_aCO_2 passed through a first order filter
ν_{O_2} (mM) Inital Value = $\nu_{O_2,n}$ = 0.05343 mM
  $[O_{2,c}]$ passed through a first order filter
ν_{P_a} (mmHg) Inital Value = $\nu_{P_a,n}$ = 43.31 mmHg
  ABP passed through a first order filter
ν_u (dimensionless) Inital Value = $\nu_{u,n}$ = 1 (dimensionless)

the demand parameter u passed through a first order filter

$[\mathrm{HbO_{2,v}}]$ (mM) Inital Value = $[\mathrm{HbO_{2,v}}]_{\mathrm{init}}$ = 2.684 mM

the concentration of bound oxygen in the veins

3.4 Temporary Variables

$$\mathrm{Cu_{A,r}} = [\mathrm{CCO}]_{\mathrm{mit}} - \mathrm{Cu_{A,o}}, \quad (\mathrm{mM}) \tag{42}$$

the concentration of reduced cytochrome-c-oxidase in the mitochondria

$$\mathrm{AVR} = \frac{V_{\mathrm{a}}}{V_{\mathrm{v}}}, \quad (\mathrm{dimensionless}) \tag{43}$$

arterio-venous volume ratio

$$\mathrm{cyt\ a_{3,o}} = [\mathrm{CCO}]_{\mathrm{mit}} - \mathrm{cyt\ a_{3,r}}, \quad (\mathrm{mM}) \tag{44}$$

concentration of oxidised cytochrome a_3 in mitochondria

$$C_{0i,m} = \frac{10^{-\mathrm{pH_m}} - 10^{-\mathrm{pH_m}-\mathrm{dpH}}}{\mathrm{dpH}}, \quad (\mathrm{dimensionless}) \tag{45}$$

natural buffering capacity for protons in mitochondria

$$C_{0i,c} = \frac{10^{-\mathrm{pH_o}} - 10^{-\mathrm{pH_o}-\mathrm{dpH}}}{\mathrm{dpH}}, \quad (\mathrm{dimensionless}) \tag{46}$$

natural buffering capacity for protons in the cytoplasm

$$\mathrm{CBF} = G\left(P_{a2} - P_{v}\right), \quad (\mathrm{ml_{blood}\,ml_{brain}}^{-1}\,\mathrm{s}^{-1}) \tag{47}$$

cerebral blood flow

$$\Delta\mathrm{oxCCO} = \Delta\mathrm{oxCCO_{offset}} + 1000V_{\mathrm{mit}}\left(\left(1 - d_f\right)\mathrm{Cu_{A,o}} - \mathrm{Cu_{A,o,n}}\right), \quad (\mu\mathrm{M}) \tag{48}$$

the expected CCO signal (as measured by NIRS)

$$\mathrm{CMR_{gluc}} = T_{\mathrm{Gluc_{in}}} - T_{\mathrm{Gluc_{out}}}, \quad (\mathrm{mM\,s^{-1}}) \tag{49}$$

rate of glucose metabolism

$$\mathrm{CMR_{lac}} = T_{\mathrm{Lac_{in}}} - T_{\mathrm{Lac_{out}}}, \quad (\mathrm{mM\,s^{-1}}) \tag{50}$$

rate of lactate metabolism

$$\mathrm{CMRO_2} = \left(1 - d_f\right) f_3 V_{\mathrm{mit}}, \quad (\mathrm{mM\,s^{-1}}) \tag{51}$$

cerebral metabolic rate of oxygen consumption

$$C_{\mathrm{NADH}} = \frac{Z}{2}\log\left(\frac{1}{[\mathrm{NAD}]/[\mathrm{NADH}]}\right), \quad (\mathrm{mV}) \tag{52}$$

redox potential minus standard redox potential for NADH at normal demand

$$\Delta G_1 = -4\left(E_1 + Z\log\left(\frac{\mathrm{Cu_{A,o}}}{\mathrm{Cu_{A,r}}}\right)\right) + p_1\Delta\mathrm{p}, \quad (\mathrm{mV}) \tag{53}$$

free energy associated with reaction ared

$$\Delta G_2 = -4\left(E_2 + Z\left(\log\left(\frac{\mathrm{Cu_{A,r}}}{\mathrm{Cu_{A,o}}}\right) - \log\left(\frac{\mathrm{cyt\ a_{3,r}}}{\mathrm{cyt\ a_{3,o}}}\right)\right)\right) + p_2\Delta\mathrm{p}, \quad (\mathrm{mV}) \tag{54}$$

free energy associated with reaction aox

$$\Delta\mathrm{G} = \Delta\mathrm{G}^{\circ} + ZF\log\left(g_p\right), \quad (\mathrm{J\,mol^{-1}}) \tag{55}$$

Gibbs free energy of ATP hydrolysis

$$\Delta\mathrm{Hbdiff} = \Delta\mathrm{Hbdiff_{offset}} + \Delta\mathrm{HbO_2} - \Delta\mathrm{HHb}, \quad (\mu\mathrm{M}) \tag{56}$$

change in ($HbO_2 - HHb$) (NIRS)

$$\Delta\mathrm{HbO_2} = \Delta\mathrm{HbO_{2,offset}} + \mathrm{HbO_2} - \mathrm{HbO_{2,n}}, \quad (\mu\mathrm{M}) \tag{57}$$

change in total oxygenated haemoglobin (NIRS)

$$\Delta\mathrm{Hbt} = \Delta\mathrm{HbT_{offset}} + \mathrm{Hbt} - \mathrm{Hbt_n}, \quad (\mu\mathrm{M}) \tag{58}$$

change in total haemoglobin (NIRS)

$$\Delta\mathrm{HHb} = \Delta\mathrm{HHb_{offset}} + \mathrm{HHb} - \mathrm{HHb_n}, \quad (\mu\mathrm{M}) \tag{59}$$

change in total deoxygenated haemoglobin (NIRS)

$$\Delta\mathrm{p} = \Delta\Psi + Z\Delta\mathrm{pH}, \quad (\mathrm{mV}) \tag{60}$$

the proton motive force across the mitochondrial inner membrane

$$\Delta\mathrm{pH} = \mathrm{pH_m} - \mathrm{pH_o}, \quad (\mathrm{dimensionless}) \tag{61}$$

the pH difference across mitochondrial inner membrane

$$E_1 = E_{1,NADH}, \quad (\mathrm{mV}) \tag{62}$$

the energy provided by electron transfer to $\mathrm{Cu_{A,o}}$

$$E_{1,NADH} = \mathcal{E}_0(\mathrm{Cu_A}) - \mathcal{E}_0(\mathrm{NADH}) + C_{\mathrm{NADH}}, \quad (\mathrm{mV}) \tag{63}$$

E_1 when the reducing substrate is NADH

$$\eta = R_P\left(\frac{\nu_{P_a}}{\nu_{P_a,n}} - 1\right) + R_O\left(\frac{\nu_{\mathrm{O_2}}}{\nu_{\mathrm{O_2},n}} - 1\right) + R_u\left(1 - \frac{\nu_u}{\nu_{u,n}}\right) + R_C\left(1 - \frac{\nu_{\mathrm{CO_2}}}{\nu_{\mathrm{CO_2},n}}\right), \quad \text{(dimensionless)} \tag{64}$$

total autoregulatory stimuli

$$f_1 = \mathrm{k_1 Cu_{A,o}} - \mathrm{k_{-1} Cu_{A,r}}, \quad (\mathrm{mM\,s^{-1}}) \tag{65}$$

the rate at which $\mathrm{Cu_{A,o}}$ is reduced

$$f_2 = \mathrm{k_2 Cu_{A,r} cyt\ a_{3,o}} - \mathrm{k_{-2} Cu_{A,o} cyt\ a_{3,r}}, \quad (\mathrm{mM\,s^{-1}}) \tag{66}$$

the rate at which $\mathrm{Cu_{A,r}}$ is oxidised and $\mathrm{cyt\ a_{3,o}}$ reduced

$$f_3 = \frac{k_3 [\mathrm{O_2}] \mathrm{cyt\ a_{3,r}} \exp\left(-c3\left(\Delta\mathrm{p} - \Delta\mathrm{p_{30}}\right)\right)}{1 + \exp\left(-c3\left(\Delta\mathrm{p} - \Delta\mathrm{p_{30}}\right)\right)}, \quad (\mathrm{mM\,s^{-1}}) \tag{67}$$

the rate at which $\mathrm{cyt\ a_{3,r}}$ is oxidised

$$G = K_G r^4, \quad (\mathrm{ml_{blood}\,ml_{brain}}^{-1}\,\mathrm{mmHg}^{-1}\,\mathrm{s}^{-1}) \tag{68}$$

resistance of cerebral circulation

$$g_p = \frac{[\mathrm{ADP}][\mathrm{P_i}]}{1000[\mathrm{ATP}]}, \quad \text{(dimensionless)} \tag{69}$$

(normalised) phophorylation potential (or ADP/ATP ratio)

$$G_t = \frac{G G_0 G_v}{G G_0 + G G_v + G_v G_0}, \quad (\mathrm{ml_{blood}\,ml_{brain}}^{-1}\,\mathrm{mmHg}^{-1}\,\mathrm{s}^{-1}) \tag{70}$$

the total conductance of the cerebral blood vessels

$$h = -r + \sqrt{r^2 + 2 r_0 h_0 + {h_0}^2}, \quad (\mathrm{cm}) \tag{71}$$

wall thickness of cerebral vessels

$$\mathrm{HbO_2} = 1000 \frac{V_\mathrm{a}[\mathrm{HbO_{2,a}}] + V_\mathrm{v}[\mathrm{HbO_{2,v}}]}{4} V_{\mathrm{blood,n}}, \quad (\mu\mathrm{M}) \tag{72}$$

total oxygenated haemoglobin (NIRS)

$$\mathrm{Hbt} = 1000 \frac{\left(V_\mathrm{a} + V_\mathrm{v}\right)[\mathrm{Hbtot}]}{4} V_{\mathrm{blood,n}}, \quad (\mu\mathrm{M}) \tag{73}$$

total haemoglobin (NIRS)

$$\mathrm{HHb} = 1000 \frac{V_\mathrm{a}[\mathrm{HHb_a}] + V_\mathrm{v}[\mathrm{HHb_v}]}{4} V_{\mathrm{blood,n}}, \quad (\mu\mathrm{M}) \tag{74}$$

total deoxygenated haemoglobin (NIRS)

$$J_{\mathrm{O2,min}} = \min\left(D_{\mathrm{O2}}\left([\mathrm{O_{2,c}}] - [\mathrm{O_2}]\right)\mathrm{CBF}[\mathrm{HbO_{2,a}}]\right), \quad (\mathrm{mM\,s^{-1}}) \tag{75}$$

rate at which oxygen is supplied to the mitochondria, minimum of diffusion rate and delivery rate

$$J_{\mathrm{O2}} = J_{\mathrm{O2,min}}, \quad (\mathrm{mM\,s^{-1}}) \tag{76}$$

the rate at which oxygen is supplied to the mitochondria

$$\mathrm{k_1} = \mathrm{k_{1,0}}\exp\left(-c_{k1}\left(\Delta\mathrm{p} - \Delta\mathrm{p_n}\right)\right), \quad (\mathrm{s^{-1}}) \tag{77}$$

forward rate constant for reaction ared

$$\mathrm{k_{1,0}} = \frac{\mathrm{k_{1,n}}\dfrac{\mathrm{N_t}}{1 + [\mathrm{NAD}]/[\mathrm{NADH}]}}{\mathrm{NADH_n}}, \quad (\mathrm{s^{-1}}) \tag{78}$$

forward rate constant for reaction ared at normal $\Delta\mathrm{p}$

$$\mathrm{k_2} = \mathrm{k_{2,n}}\exp\left(-c_{k2}\left(\Delta\mathrm{p} - \Delta\mathrm{p_n}\right)\right), \quad (\mathrm{mM^{-1}\,s^{-1}}) \tag{79}$$

forward rate constant for reaction aox

$$\mathrm{k_{ATP,C_V}} = \frac{L_{CV}V_{\mathrm{mit}}}{n_a}, \quad (\mathrm{mM\,s^{-1}}) \tag{80}$$

rate of ATP synthesis by Complex V

$$\mathrm{Keq_1} = 10^{(-1/Z)(p_1\Delta\mathrm{p}/4 - E_1)}, \quad (\mathrm{dimensionless}) \tag{81}$$

equilibrium constant for reaction ared

$$\mathrm{Keq_2} = 10^{(-1/Z)(p_2\Delta\mathrm{p}/4 - E_2)}, \quad (\mathrm{dimensionless}) \tag{82}$$

equilibrium constant for reaction aox

$$k_{\mathrm{MAshut}} = \frac{\mathrm{CMRO_{2,n}}[\mathrm{NADH_{cyt}}]}{3\left([\mathrm{NADH_{cyt}}]_\mathrm{n}[\mathrm{NAD}]_\mathrm{n}[\mathrm{H^+}]_\mathrm{n} - \dfrac{1}{\mathrm{Keq_{MAshut}}}[\mathrm{NAD_{cyt}}]_\mathrm{n}\mathrm{NADH_n}\mathrm{H^+_{m,n}}\right)}, \quad (\mathrm{mM^{-1}\,s^{-1}}) \tag{83}$$

rate of forward reaction in the malate-aspartate shuttle

$$\mathrm{k_{-1}} = \frac{\mathrm{k_1}}{\mathrm{Keq_1}}, \quad (\mathrm{s^{-1}}) \tag{84}$$

backward rate constant for reaction ared

$$\mathrm{k_{-2}} = \frac{\mathrm{k_2}}{\mathrm{Keq_2}}, \quad (\mathrm{mM^{-1}\,s^{-1}}) \tag{85}$$

backward rate constant for reaction aox

$$k_{\mathrm{MAshut}}^{-} = \frac{k_{\mathrm{MAshut}}[\mathrm{NADH}]}{[\mathrm{NADH_{cyt}}]\mathrm{Keq_{MAshut}}}, \quad (\mathrm{mM}^{-1}\,\mathrm{s}^{-1}) \tag{86}$$

rate of backward reaction in the malate-aspartate shuttle

$$k_{\mathrm{pl}} = \frac{2\mathrm{CMR_{gluc,n}} - \dfrac{\mathrm{CMRO_{2,n}}}{3} + k_{\mathrm{pl}}^{-}[\mathrm{lac}]_{\mathrm{n}}[\mathrm{NAD_{cyt}}]_{\mathrm{n}}}{[\mathrm{Py}]_{\mathrm{n}}[\mathrm{H}^{+}]_{\mathrm{n}}} \frac{[\mathrm{NADH_{cyt}}]}{[\mathrm{NADH_{cyt}}]_{\mathrm{n}}}, \quad (\mathrm{mM}^{-1}\,\mathrm{s}^{-1}) \tag{87}$$

rate of forward reaction in the pyruvate lactate equilibrium

$$k_{\mathrm{TCA}} = \frac{v_{\mathrm{TCA}}[\mathrm{Py}][\mathrm{NAD}]}{\left(k_{m,\mathrm{tcaN}} + [\mathrm{NAD}]\right)\left(k_{m,\mathrm{tcaP}} + [\mathrm{Py}]\right)}, \quad (\mathrm{mM}\,\mathrm{s}^{-1}) \tag{88}$$

rate of the TCA cycle

$$L = L_{CV} + L_{lk}, \quad (\mathrm{mM}\,\mathrm{s}^{-1}) \tag{89}$$

the rate at which protons reenter the mitochondrial matrix

$$L_{CV} = \frac{CV_{inh}L_{CV,\mathrm{max}}\left(1 - e^{-\theta}\right)}{1 + r_{CV}e^{-\theta}}, \quad (\mathrm{mM}\,\mathrm{s}^{-1}) \tag{90}$$

the rate at which protons reenter the mitochondrial matrix associated with ADP phosphorylation

$$L_{lk} = k_{\mathrm{unc}}L_{lk0}\left(\exp\left(\Delta\mathrm{p}k_{lk2}\right) - 1\right), \quad (\mathrm{mM}\,\mathrm{s}^{-1}) \tag{91}$$

the rate at which protons reenter the mitochondrial matrix through leak channels

$$\mu = \frac{\mu_{\mathrm{min}} + \mu_{\mathrm{max}}e^{\eta}}{1 + e^{\eta}}, \quad (\mathrm{dimensionless}) \tag{92}$$

total autoregulatory stimuli filtered through sigmoidal function

$$[\mathrm{NADH}] = \mathrm{N_t} - [\mathrm{NAD}], \quad (\mathrm{mM}) \tag{93}$$

concentration of NADH in the mitochondria

$$[\mathrm{NADH_{cyt}}] = [\mathrm{NAD_{cyt}}]_{\mathrm{n}} + [\mathrm{NADH_{cyt}}]_{\mathrm{n}} - [\mathrm{NAD_{cyt}}], \quad (\mathrm{mM}) \tag{94}$$

concentration of NADH in the cytoplasm

$$[\mathrm{NAD}]/[\mathrm{NADH}] = \frac{[\mathrm{NAD}]}{[\mathrm{NADH}]}, \quad (\mathrm{dimensionless}) \tag{95}$$

NAD/NADH ratio

$$\mathrm{NTP/EPP} = \frac{\left(1 - d_f\right)[\mathrm{ATP}]}{\mathrm{EPP}}, \quad (\mathrm{dimensionless}) \tag{96}$$

the ratio [ATP]/EPP

$$P_1 = \frac{P_{a2} + P_v}{2}, \quad \text{(mmHg)} \tag{97}$$

average blood pressure in vessels

$$P_{a2} = \frac{G_0 P_a + G P_v}{G + G_0}, \quad \text{(mmHg)} \tag{98}$$

the pressure at the start of the cerebral artery compartment

$$\mathrm{PCr/EPP} = \frac{\left(1 - d_f\right)[\mathrm{PCr}]}{\mathrm{EPP}}, \quad \text{(dimensionless)} \tag{99}$$

the ratio [PCr]/EPP

$$\mathrm{pH_m} = -\log\left(\frac{[\mathrm{H_m^+}]}{1000}\right), \quad \text{(dimensionless)} \tag{100}$$

mitochondrial pH

$$\mathrm{pH_o} = -\log\left(\frac{[\mathrm{H_{cyt}^+}]}{1000}\right), \quad \text{(dimensionless)} \tag{101}$$

extra-mitochondrial pH

$$\mathrm{P_i/EPP} = \frac{\left(1 - d_f\right)[\mathrm{P_i}]}{\mathrm{EPP}} + d_f, \quad \text{(dimensionless)} \tag{102}$$

the ratio [Pi]/EPP

$$P_v = \frac{G_t}{G_v}\left(P_a - P_{vs}\right) + P_{vs}, \quad \text{(mmHg)} \tag{103}$$

venous blood pressure

$$R_{\mathrm{ATPtoADP}} = \frac{[\mathrm{ATP}]}{k_m + [\mathrm{ATP}]}, \quad \text{(dimensionless)} \tag{104}$$

Relative rate of reaction ATPtoADP

$$\mathrm{r_{buffi,m}} = \frac{\mathrm{C_{buffi,m}}}{C_{0i,m}}, \quad \text{(dimensionless)} \tag{105}$$

buffering capacity for protons in mitochondria

$$\mathrm{r_{buffi,c}} = \frac{\mathrm{C_{buffi,c}}}{C_{0i,c}}, \quad \text{(dimensionless)} \tag{106}$$

buffering capacity for protons in the cytoplasm

$$R_{\mathrm{Gluc_{in}}} = \mathrm{V_{glucosein}}, \quad \text{(dimensionless)} \tag{107}$$

Relative rate of reaction $\mathrm{Gluc_{in}}$

$$R_{\mathrm{Gluc_{out}}} = \frac{[\mathrm{gluc}]}{k_{\mathrm{glut}} + [\mathrm{gluc}]}, \quad \text{(dimensionless)} \tag{108}$$

Relative rate of reaction $\mathrm{Gluc_{out}}$

$$R_{\mathrm{Glycolysis}} = \frac{[\mathrm{ADP}]^2[\mathrm{P_i}]^2[\mathrm{gluc}][\mathrm{NAD_{cyt}}]^2}{\left(k_{m,\mathrm{glycA}}{}^2 + [\mathrm{ADP}]^2\right)\left(k_{m,\mathrm{glycP}}{}^2 + [\mathrm{P_i}]^2\right)\left(k_{m,\mathrm{glycG}} + [\mathrm{gluc}]\right)\left(k_{m,\mathrm{glycN}}{}^2 + [\mathrm{NAD_{cyt}}]^2\right)}, \quad \text{(dimensionless)} \tag{109}$$

Relative rate of reaction Glycolysis

$$\mathrm{R_{Hi}} = \mathrm{r_{buffi,m}}, \quad \text{(dimensionless)} \tag{110}$$

relative mitochondrial volume for protons

$$\mathrm{R_{Hi,c}} = \mathrm{r_{buffi,c}}, \quad \text{(dimensionless)} \tag{111}$$

relative cytoplasmic volume for protons

$$R_{\mathrm{Lac_{in}}} = \mathrm{V_{lacin}}, \quad \text{(dimensionless)} \tag{112}$$

Relative rate of reaction $\mathrm{Lac_{in}}$

$$R_{\mathrm{Lac_{out}}} = \frac{[\mathrm{lac}][\mathrm{H^+_{cyt}}]}{\left(k_{\mathrm{MCT}} + [\mathrm{lac}]\right)\left(k_{\mathrm{MCT,H^+}} + [\mathrm{H^+_{cyt}}]\right)}, \quad \text{(dimensionless)} \tag{113}$$

Relative rate of reaction $\mathrm{Lac_{out}}$

$$\mathrm{ScO_2} = \frac{\mathrm{SaO_2} + \mathrm{SvO2}}{2}, \quad \text{(dimensionless)} \tag{114}$$

capillary oxygen saturation

$$\sigma_e = \sigma_{e0}\left(\exp\left(\frac{K_\sigma\,(r - r_0)}{r_0}\right) - 1\right) - \sigma_{\mathrm{coll}}, \quad \text{(mmHg)} \tag{115}$$

elastic stress in vessel walls

$$\mathrm{SvO2} = \frac{[\mathrm{HbO_{2,v}}]}{[\mathrm{Hbtot}]}, \quad \text{(dimensionless)} \tag{116}$$

venous oxygen saturation

$$T_{\mathrm{AK}} = k_{\mathrm{AK}}[\mathrm{ADP}]^2 - k^-_{\mathrm{AK}}[\mathrm{ATP}][\mathrm{AMP}], \quad (\mathrm{mM\,s^{-1}}) \tag{117}$$

Rate of reaction AK

$$T_{\mathrm{ADPtoATP}} = \mathrm{k_{ATP,C_V}}, \quad (\mathrm{mM\,s^{-1}}) \tag{118}$$

Rate of reaction ADPtoATP

$$T_{\mathrm{aox}} = f_2, \quad (\mathrm{mM\,s^{-1}}) \tag{119}$$

Rate of reaction aox

$$T_{\mathrm{ared}} = f_1, \quad (\mathrm{mM\,s^{-1}}) \tag{120}$$

Rate of reaction ared

$$T_{\mathrm{ATPtoADP}} = \frac{\mathrm{V}_{\mathrm{max,ATP}}[\mathrm{ATP}]}{k_m + [\mathrm{ATP}]}, \quad (\mathrm{mM\,s^{-1}}) \tag{121}$$

Rate of reaction ATPtoADP

$$T_{\mathrm{box}} = f_3, \quad (\mathrm{mM\,s^{-1}}) \tag{122}$$

Rate of reaction box

$$T_e = \sigma_e h, \quad (\mathrm{mmHg\,cm}) \tag{123}$$

elastic tension in vessel walls

$$T_{\mathrm{Gluc_{in}}} = v_{\mathrm{glut}} \mathrm{V}_{\mathrm{glucosein}}, \quad (\mathrm{mM\,s^{-1}}) \tag{124}$$

Rate of reaction $\mathrm{Gluc_{in}}$

$$T_{\mathrm{Gluc_{out}}} = \frac{v_{\mathrm{glut}}[\mathrm{gluc}]}{k_{\mathrm{glut}} + [\mathrm{gluc}]}, \quad (\mathrm{mM\,s^{-1}}) \tag{125}$$

Rate of reaction $\mathrm{Gluc_{out}}$

$$T_{\mathrm{Glycolysis}} = \frac{v_{\mathrm{glyc}}[\mathrm{ADP}]^2[\mathrm{P_i}]^2[\mathrm{gluc}][\mathrm{NAD_{cyt}}]^2}{\left({k_{m,\mathrm{glycA}}}^2 + [\mathrm{ADP}]^2\right)\left({k_{m,\mathrm{glycP}}}^2 + [\mathrm{P_i}]^2\right)\left(k_{m,\mathrm{glycG}} + [\mathrm{gluc}]\right)\left({k_{m,\mathrm{glycN}}}^2 + [\mathrm{NAD_{cyt}}]^2\right)}, \quad (\mathrm{mM\,s^{-1}}) \tag{126}$$

Rate of reaction Glycolysis

$$\theta = k_{CV}\left(\Delta \mathrm{p} + \frac{Z}{n_a}\log\left(\frac{g_p}{g_{p,\mathrm{n}}}\right) - \Delta \mathrm{p_{CV0}}\right), \quad (\text{dimensionless}) \tag{127}$$

driving force for Complex V

$$T_{\mathrm{Lac_{in}}} = v_{\mathrm{MCT}} \mathrm{V}_{\mathrm{lacin}}, \quad (\mathrm{mM\,s^{-1}}) \tag{128}$$

Rate of reaction $\mathrm{Lac_{in}}$

$$T_{\mathrm{Lac_{out}}} = \frac{v_{\mathrm{MCT}}[\mathrm{lac}][\mathrm{H^+_{cyt}}]}{\left(k_{\mathrm{MCT}} + [\mathrm{lac}]\right)\left(k_{\mathrm{MCT,H^+}} + [\mathrm{H^+_{cyt}}]\right)}, \quad (\mathrm{mM\,s^{-1}}) \tag{129}$$

Rate of reaction $\mathrm{Lac_{out}}$

$$T_m = T_{\max} \exp\left(-\left(\left|\frac{r - r_m}{r_t - r_m}\right|\right)^{n_m}\right), \quad (\mathrm{mmHg\,cm}) \tag{130}$$

muscular tension in vessel walls

$$T_{\mathrm{MAshuttle}} = k_{\mathrm{MAshut}}[\mathrm{H}^+_{\mathrm{cyt}}][\mathrm{NAD}] - k^-_{\mathrm{MAshut}}[\mathrm{NAD_{cyt}}][\mathrm{H}^+_{\mathrm{m}}], \quad (\mathrm{mM\,s^{-1}}) \tag{131}$$

Rate of reaction MAshuttle

$$T_{\max} = T_{\max 0}\left(1 + k_{\mathrm{aut}}\mu\right), \quad (\mathrm{mmHg\,cm}) \tag{132}$$

maximum muscular tension developed by circulation

$$T_{\mathrm{O_2in}} = J_{\mathrm{O2}}, \quad (\mathrm{mM\,s^{-1}}) \tag{133}$$

Rate of reaction O_2in

$$\mathrm{TOS} = \frac{100}{[\mathrm{Hbtot}]}\left(\frac{\left(\frac{r}{r_n}\right)^2[\mathrm{HbO_{2,a}}]}{\left(\frac{r}{r_n}\right)^2 + \frac{V_{\mathrm{v}}}{V_{\mathrm{a,n}}}} + \frac{\frac{V_{\mathrm{v}}}{V_{\mathrm{a,n}}}[\mathrm{HbO_{2,v}}]}{\left(\frac{r}{r_n}\right)^2 + \frac{V_{\mathrm{v}}}{V_{\mathrm{a,n}}}}\right), \quad (\mathrm{dimensionless}) \tag{134}$$

tissue oxygenation index

$$T_{\mathrm{CK}} = k_{\mathrm{PCr}}[\mathrm{PCr}][\mathrm{ADP}][\mathrm{H}^+_{\mathrm{cyt}}] - k^-_{\mathrm{PCr}}[\mathrm{ATP}][\mathrm{Cr}], \quad (\mathrm{mM\,s^{-1}}) \tag{135}$$

Rate of reaction CK

$$T_{\mathrm{psiout}} = L, \quad (\mathrm{mM\,s^{-1}}) \tag{136}$$

Rate of reaction psiout

$$T_{\mathrm{PytoLac}} = k_{\mathrm{pl}}[\mathrm{Py}][\mathrm{H}^+_{\mathrm{cyt}}] - k^-_{\mathrm{pl}}[\mathrm{lac}][\mathrm{NAD_{cyt}}], \quad (\mathrm{mM\,s^{-1}}) \tag{137}$$

Rate of reaction PytoLac

$$T_{\mathrm{TCA}} = k_{\mathrm{TCA}}, \quad (\mathrm{mM\,s^{-1}}) \tag{138}$$

Rate of reaction TCA

$$\mathrm{V_{max,ATP}} = \left(\frac{L_{CV,\mathrm{n}}V_{\mathrm{mit}}}{n_a} + 2\mathrm{CMR_{gluc,n}}\right)\left(1 + k_{m,\mathrm{ATP}}\right)u, \quad (\mathrm{mM\,s^{-1}}) \tag{139}$$

Vmax of ATP use

$$v_{\mathrm{glyc}} = \frac{v_{\mathrm{glyc,n}}\left(I + 1\right)}{1 + I\frac{[\mathrm{ATP}]}{[\mathrm{ATP}]_{\mathrm{n}}}\frac{[\mathrm{AMP}]_{\mathrm{n}}}{[\mathrm{AMP}]}}, \quad (\mathrm{mM\,s^{-1}}) \tag{140}$$

Vmax for glycolysis

$$\mathrm{V_{mca}} = \mathrm{CBFCBFscale}, \quad (\mathrm{cm\,s^{-1}}) \tag{141}$$

the velocity of blood in the middle cerebral artery

$$V_{\mathrm{a}} = V_{\mathrm{a,n}}\left(\frac{r}{r_n}\right)^2, \quad \text{(dimensionless)} \tag{142}$$

arterial blood volume as a fraction of normal total blood volume

$$V_{\mathrm{t}} = V_{\mathrm{a}} + V_{\mathrm{v}}, \quad \text{(dimensionless)} \tag{143}$$

normalised total blood volume

$$V_{\mathrm{v}} = V_{\mathrm{v,n}} + C_v\left(P_v - P_{v,\mathrm{n}}\right), \quad \text{(dimensionless)} \tag{144}$$

venous blood volume as a fraction of total blood volume

$$[\mathrm{HHb_v}] = [\mathrm{Hbtot}] - [\mathrm{HbO_{2,v}}], \quad \text{(mM)} \tag{145}$$

the concentration of deoxyhaemoglobin in the veins

3.5 Parameters

$[\mathrm{ADP}]_\mathrm{n} = 0.0120\,\mathrm{mM}$ Range: 0.0096 to 0.0144
: the normal concentration of ADP in the cytoplasm [1]

$\mathrm{Cu_{A,frac,n}} = 0.67$ (dimensionless) Range: 0.00 to 1.00
: normal oxidised fraction of $\mathrm{Cu_A}$

$[\mathrm{ATP}]_\mathrm{n} = 1.2\,\mathrm{mM}$ Range: 0.1 to 10.0
: the normal concentration of ATP in the cytoplasm [2]

$V_{\mathrm{blood,n}} = 0.0325$ (dimensionless) Range: 0.0260 to 0.0390
: normal blood volume as a fraction of brain tissue volume

$c3 = 0.110\,\mathrm{mV^{-1}}$ Range: 0.088 to 0.120
: parameter controlling the sensitivity of reaction box to $\Delta\mathrm{p}$

$\mathrm{CBF_n} = 0.0080\,\mathrm{ml_{blood}\,ml_{brain}}^{-1}\,\mathrm{s^{-1}}$ Range: 0.0064 to 0.0096
: normal cerebral blood flow

CBFscale = 5000 cm Not included in sensitivity analysis
: ratio between $\mathrm{V_{mca}}$ and CBF

$\mathrm{C_{buffi,m}} = 0.0220$ (dimensionless) Range: 0.0176 to 0.0264
: buffering capacity for protons in mitochondria

$\mathrm{C_{buffi,c}} = 0.025$ (dimensionless) Range: 0.020 to 0.030
: buffering capacity for protons in the cytoplasm

$\Delta\mathrm{oxCCO_{offset}} = 0\,\mu\mathrm{M}$ Not included in sensitivity analysis
: an arbitrary baseline offset to the ΔoxCCO signal (NIRS)

$\mathrm{occ_{frac}} = 0.80$ (dimensionless) Range: 0.64 to 0.96
: fraction of arterial blood which flows through the carotid arteries under normal conditions

$C_{im} = 0.00675\,\mathrm{mM\,mV^{-1}}$ Range: 0.00540 to 0.00810
: capacitance of mitochondrial inner membrane

$c_{k1} = 0.010\,\mathrm{mV^{-1}}$ Range: 0.008 to 0.012
: parameter controlling sensitivity of $\mathrm{k_1}$ to $\Delta\mathrm{p}$

$c_{k2} = 0.020\,\mathrm{mV^{-1}}$ Range: 0.016 to 0.024

parameter controlling sensitivity of k_2 to Δp

$CMR_{gluc,n} = 0.00440\,mM\,s^{-1}$ Range: 0.00352 to 0.00528

normal rate of glucose metabolism for the brain [3]

$CMRO_{2,n} = 0.020\,mM\,s^{-1}$ Range: 0.016 to 0.024

The resting $CMRO_2$

$C_v = 0.0470\,mmHg^{-1}$ Range: 0.0376 to 0.0564

compliance of the veins (normalised)

$CV_{inh} = 1.0$ (dimensionless) Range: 0.8 to 1.2

a control parameter representing the action of Complex V inhibitors

$[CCO]_{tis} = 0.0022\,mM$ Range: 0.0010 to 0.0070

concentration of cytochrome c oxidase in tissue

$d = 0$ (dimensionless) Not included in sensitivity analysis

a parameter controlling the fraction of cells considered as dead

$\Delta Hbdiff_{offset} = 0\,\mu M$ Not included in sensitivity analysis

an arbitrary baseline offset to the $\Delta Hbdiff$ signal (NIRS)

$\Delta HbO_{2,offset} = 0\,\mu M$ Not included in sensitivity analysis

an arbitrary baseline offset to the ΔHbO_2 signal (NIRS)

$\Delta HbT_{offset} = 0\,\mu M$ Not included in sensitivity analysis

an arbitrary baseline offset to the ΔHbt signal (NIRS)

$\Delta HHb_{offset} = 0\,\mu M$ Not included in sensitivity analysis

an arbitrary baseline offset to the ΔHHb signal (NIRS)

$\Delta p_{3,corr} = -25\,mV$ Range: -28 to -20

Δp_{30} minus normal Δp

$dpH = 0.0010$ (dimensionless) Range: 0.0008 to 0.0012

a constant in the buffering relationship

$\Delta\Psi_n = 145\,mV$ Range: 125 to 150

normal mitochondrial inner membrane potential

$\mathcal{E}_0(\text{cyt } a_3) = 350\,mV$ Range: 280 to 420

cytochrome a_3 standard redox potential

$\mathcal{E}_0(Cu_A) = 247.0\,mV$ Range: 197.6 to 250.0

Cu_A standard redox potential

$\mathcal{E}_0(NADH) = -320\,mV$ Range: -384 to -256

NADH standard redox potential

$F = 96.48\,C\,mmol^{-1}$ Not included in sensitivity analysis

Faraday constant

$G_{0,frac} = 5$ (dimensionless) Range: 4 to 6

ratio between the conductance of the cerebral arteries and the supplying artery compartment

$[gluc_c] = 5.30\,mM$ Range: 4.24 to 6.36

concentration of glucose in the blood [4]

$[gluc]_n = 1.20\,mM$ Range: 0.96 to 1.44

normal cellular concentration of glucose [5]

$\Delta G^\circ = -3.05e+04\,J\,mol^{-1}$ Range: -3.07e+04 to -3.03e+04

standard Gibbs free energy of ATP hydrolysis

$G_{VArat,n} = 4.0$ (dimensionless) Range: 3.2 to 4.8

normal ratio of conductances between arteries and veins G_v/G_n (determines venous pressure) [6]

$h_0 = 0.0030\,cm$ Range: 0.0024 to 0.0036

vascular wall thickness when radius is r_0

$k_{H,O_2} = 0.0014\,\mathrm{mM\,mmHg^{-1}}$ Not included in sensitivity analysis
constant setting relationship between oxygen saturation and oxygen concentration in artery

$I = 3$ (dimensionless) Range: 0 to 20
the parameter which describes how strongly the AMP/ATP ratio inhibits the conversion of glucose to pyruvate

$k_{3,0} = 2.5e+05\,\mathrm{mM^{-1}\,s^{-1}}$ Range: 2.0e+05 to 3.0e+05
an apparent second-order rate constant for reaction box at zero Δp

$k_{AK} = 1055\,\mathrm{mM^{-1}\,s^{-1}}$ Range: 844 to 1266
the forward rate constant for the conversion of two molecules of ADP to one of ATP and one of AMP [7, 8].

$k_{aut} = 1$ (dimensionless) Range: 0 to 1
control parameter allowing destruction of autoregulation

$\mathrm{Keq}_{MAshut} = 10$ (dimensionless) Range: 8 to 12
equilibrium constant for the malate-aspartate shuttle

$k_{glut} = 6.20\,\mathrm{mM}$ Range: 4.96 to 7.44
k_m for the transport of glucose in and out of the cell [9]

$k_{MCT,H^+} = 0.0000\,\mathrm{mM}$ Range: 0.0000 to 0.0002
k_m for the transport of a proton coupled with lactate in and out of the cell

$k_{MCT} = 2.0\,\mathrm{mM}$ Range: 1.6 to 2.4
k_m for the transport of lactate in and out of the cell

$k_{lk2} = 0.0380\,\mathrm{mV^{-1}}$ Range: 0.0304 to 0.0456
second constant controlling rate of L_{lk} of Δp

$k_{m,ATP} = 0.025$ (dimensionless) Range: 0.020 to 0.030
k_m for ATP use as a fraction of normal ATP concentration [7]

$k_{m,glycA,f} = 0.20$ (dimensionless) Range: 0.16 to 0.24
k_m for ADP in glycolysis as a fration of normal ADP concentration [8]

$k_{m,glycG} = 0.05\,\mathrm{mM}$ Range: 0.04 to 0.06
k_m for glucose in the caricature of glycolysis [10]

$k_{m,glycN} = 0.00\,\mathrm{mM}$ Range: 0.18 to 1.00
k_m for NAD in the caricature of glycolysis

$k_{m,glycP,f} = 0.20$ (dimensionless) Range: 0.16 to 0.24
k_m for inorganic phosphate in glycolysis as a fraction of normal phosphate concentration [8]

$k_{m,tcaN,f} = 0.6$ (dimensionless) Range: 0.0 to 10.0
k_m for NAD in the TCA cycle as a fraction of normal NAD concentration

$k_{m,tcaP,f} = 0.005$ (dimensionless) Range: 0.000 to 10.000
k_m for pyruvate in the TCA cycle as a fraction of normal pyruvate concentration

$k^-_{AK} = 379.0\,\mathrm{mM^{-1}\,s^{-1}}$ Range: 303.2 to 454.8
the backward rate constant for the conversion of two molecules of ADP to one of ATP and one of AMP [7, 8].

$\mathrm{K}^*_{eq,PCr} = 166.0$ (dimensionless) Range: 132.8 to 199.2

effective equilibrium constant for the reaction in which phosphocreatine combines with ADP to give creatine and ATP [11]

$t_{1/2,\mathrm{PCr}} = 2.0e - 05\,\mathrm{s}$ Range: 1.6e-05 to 2.4e-05
halftime for the reaction in which phosphocreatine combines with ADP to give creatine and ATP

$t_{1/2,\mathrm{pl}} = 10\,\mathrm{mM}^{-1}\,\mathrm{s}^{-1}$ Range: 8 to 12
time constant for pyruvate to lactate interconvertion

$K_\sigma = 10$ (dimensionless) Range: 8 to 12
parameter controlling sensitivity of σ_e to radius

$k_{\mathrm{unc}} = 1.0$ (dimensionless) Range: 0.8 to 1.2
a parameter representing the action of uncouplers

$[\mathrm{lac}]_\mathrm{n} = 3.0\,\mathrm{mM}$ Range: 0.1 to 5.0
normal concentration of lactate in the cytoplasm [12]

$[\mathrm{lac_c}] = 1.0\,\mathrm{mM}$ Range: 0.8 to 1.2
capillary lactate concentration [4]

$L_{CV,0} = 0.40$ (dimensionless) Range: 0.32 to 0.48
normal Complex V flux as a fraction of maximum possible flux

$L_{lk,\mathrm{frac}} = 0.25$ (dimensionless) Range: 0.20 to 0.30
normal fraction of proton entry into mitochondria which is via leak channels

$\mu_{\mathrm{max}} = 1$ (dimensionless) Not included in sensitivity analysis
maximum value of μ

$\mu_{\mathrm{min}} = -1$ (dimensionless) Not included in sensitivity analysis
minimum value of μ

$\mu_n = 0$ (dimensionless) Not included in sensitivity analysis
normal value of μ

$n_a = 4.33$ (dimensionless) Range: 3.80 to 5.00
number of protons passing through Complex V for each ATP synthesised [13]

$[\mathrm{NAD_{cyt}}]_\mathrm{n} = 359\,\mathrm{mM}$ Range: 318 to 400
normal concentration of NAD in the cytoplasm

$[\mathrm{NADH_{cyt}}]_\mathrm{n} = 50\,\mathrm{mM}$ Range: 32 to 68
normal concentration of NADH in the cytoplasm

$\Delta_{\mathrm{nadir}} = 0$ (dimensionless) Not included in sensitivity analysis
a switch parameter introduced to allow parameter changes at the nadir of hypoxia-ischaemia

$[\mathrm{NAD}]_\mathrm{n}/[\mathrm{NADH}]_\mathrm{n} = 9.0$ (dimensionless) Range: 0.1 to 20.0
normal NAD/NADH ratio

$\mathrm{N_t} = 3.0\,\mathrm{mM}$ Range: 2.4 to 3.6
total mitochondrial NAD and NADH concentration

$n_h = 2.5$ (dimensionless) Range: 2.0 to 3.0
Hill coefficient for haemoglobin saturation

$n_m = 1.830$ (dimensionless) Range: 1.464 to 2.196
exponent in the muscular tension relationship

$[\mathrm{O_2}]_\mathrm{n} = 0.0240\,\mathrm{mM}$ Range: 0.0192 to 0.0288
normal oxygen concentration in mitochondria

$P_a = 50\,\mathrm{mmHg}$ Range: 30 to 80
arterial blood pressure

$P_a\mathrm{CO_2} = 40\,\mathrm{mmHg}$ Range: 20 to 60
arterial partial pressure of CO_2

$P_a\mathrm{CO_{2,n}} = 40\,\mathrm{mmHg}$ Range: 32 to 48
normal arterial partial pressure of CO_2

$P_{a,\mathrm{n}} = 50\,\mathrm{mmHg}$ Range: 45 to 60
normal value of ABP

$[\mathrm{PCr}]_\mathrm{n} = 2.6\,\mathrm{mM}$ Range: 0.1 to 10.0
normal concentration of phosphocreatine in cell cytoplasm [2]

$[\mathrm{PCr}]_\mathrm{n}/[\mathrm{P_i}]_\mathrm{n} = 2.73$ (dimensionless) Range: 0.10 to 10.00
normal $\mathrm{PCr}/\mathrm{P_i}$ concentration in the cytoplasm

$\phi = 0.0360\,\mathrm{mM}$ Range: 0.0288 to 0.0432
value of O_2 concentration at half maximal saturation

$\mathrm{pH_{m,n}} = 7.4$ (dimensionless) Range: 7.2 to 7.6
normal mitochondrial pH

$\mathrm{pH_{o,n}} = 7.0$ (dimensionless) Range: 6.8 to 7.2
normal extra-mitochondrial pH

$P_{ic} = 4.5\,\mathrm{mmHg}$ Range: 3.6 to 5.4
intracranial blood pressure

$P_{ic,\mathrm{n}} = 4.5\,\mathrm{mmHg}$ Range: 3.6 to 5.4
normal intracranial blood pressure

$p_{tot} = 18.4$ (dimensionless) Range: 18.0 to 20.0
total protons pumped by reactions ared, aox and box

$P_{vs} = 1.5\,\mathrm{mmHg}$ Range: 1.2 to 1.8
pressure in the venous sinuses [14]

$[\mathrm{Py}]_\mathrm{n} = 0.10\,\mathrm{mM}$ Range: 0.08 to 0.12
normal concentration of pyruvate ions in the cytoplasm [15]

$r_0 = 0.01260\,\mathrm{cm}$ Range: 0.01008 to 0.01512
a special radius in the elastic tension relationship

$R_C = 2.2$ (dimensionless) Range: 0.0 to 10.0
parameter controlling sensitivity of η to $P_a\mathrm{CO_2}$

$R_O = 1.5$ (dimensionless) Range: 0.0 to 10.0
parameter controlling sensitivity of η to $[\mathrm{O_{2,c}}]$

$R_P = 4$ (dimensionless) Range: 0 to 10
parameter controlling sensitivity of η to P_a

$R_u = 0$ (dimensionless) Not included in sensitivity analysis
parameter controlling sensitivity of η to u

$r_{CV} = 5$ (dimensionless) Range: 4 to 6
a parameter controlling the ratio of maximal to minimal rates of oxidative phosphorylation

$r_m = 0.0270\,\mathrm{cm}$ Range: 0.0216 to 0.0324
value of vessel radius giving maximum muscular tension

$r_n = 0.01870\,\mathrm{cm}$ Range: 0.01496 to 0.02244
normal radius of blood vessels

$r_\mathrm{occ} = 0$ (dimensionless) Range: 0 to 1
fraction by which the radius of the carotid arteries has been reduced

$r_t = 0.0180\,\mathrm{cm}$ Range: 0.0144 to 0.0216
parameter in the muscular tension relationship

$\mathrm{SaO_{2,n}} = 0.96$ (dimensionless) Range: 0.90 to 1.00

normal saturation of the arterial haemoglobin

$\mathrm{SaO_2} = 0.96$ (dimensionless) Range: 0.50 to 1.00

saturation of the arterial haemoglobin

$\sigma_{\mathrm{coll}} = 62.79\,\mathrm{mmHg}$ Range: 50.23 to 75.35

value of pressure at which vessels collapse

$\sigma_{e0} = 0.1425\,\mathrm{mmHg}$ Range: 0.1140 to 0.1710

parameter in relationship determining σ_e

$\tau_{\mathrm{CO_2}} = 5\,\mathrm{s}$ Range: 4 to 6

the time constant associated with $\nu_{\mathrm{CO_2}}$

$\tau_{\mathrm{O_2}} = 20\,\mathrm{s}$ Range: 16 to 24

the time constant associated with $\nu_{\mathrm{O_2}}$

$\tau_{P_a} = 5\,\mathrm{s}$ Range: 4 to 6

the time constant associated with ν_{P_a}

$\tau_u = 0.5\,\mathrm{s}$ Range: 0.4 to 0.6

the time constant associated with ν_u

$u_n = 1$ (dimensionless) Not included in sensitivity analysis

resting "demand"

$\mathrm{VArat_n} = 3.0$ (dimensionless) Range: 2.4 to 3.6

the normal ratio of the volume of the veins to the volume of the arteries

$V_{\mathrm{mit}} = 0.0670$ (dimensionless) Range: 0.0536 to 0.0804

fraction of brain water which is mitochondria

$V_{\mathrm{t,n}} = 1.0$ (dimensionless) Range: 0.8 to 1.2

normal total blood volume

$[\mathrm{Hbtot}]_\mathrm{n} = 5.4\,\mathrm{mM}$ Range: 3.5 to 8.0

normal total haemoglobin concentration in the arteries and veins

$Z = 59.03\,\mathrm{mV}$ Not included in sensitivity analysis

$2.303\times\,\mathrm{RT}/F$ where R is the ideal gas constant ($8300\,\mathrm{mJ\,K^{-1}\,mol^{-1}}$), T is the temperature (298 K), F is the Faraday constant (9.65×10^4 $\mathrm{C\,mol^{-1}}$) and 2.303 arises from $1/\log_{10} e$.

3.6 Derived Parameters

$$\mathrm{Cu_{A,o,init}} = \mathrm{Cu_{A,o,n}} = 0.022\,\mathrm{mM} \tag{146}$$

initial oxidized $\mathrm{Cu_A}$

$$[\mathrm{AMP}]_\mathrm{n} = \frac{\mathrm{Keq_{ADPATP}}[\mathrm{ADP}]_\mathrm{n}{}^2}{[\mathrm{ATP}]_\mathrm{n}} = 0.000334\,\mathrm{mM} \tag{147}$$

normal AMP concentration in cytoplasm

$$\mathrm{Cu_{A,o,n}} = [\mathrm{CCO}]_\mathrm{mit}\mathrm{Cu_{A,frac,n}} = 0.022\,\mathrm{mM} \tag{148}$$

normal oxidized $\mathrm{Cu_A}$

$$\mathrm{Cu_{A,r,n}} = [\mathrm{CCO}]_\mathrm{mit} - \mathrm{Cu_{A,o,n}} = 0.01084\,\mathrm{mM} \tag{149}$$

the resting amount of reduced cytochrome-c-oxidase

$$\mathrm{a3_{frac,n}} = 1 - \frac{\mathrm{cyt\ a_{3,r,n}}}{\mathrm{[CCO]_{mit}}} = 0.9748\,(\mathrm{dimensionless}) \tag{150}$$

normal oxidised fraction of cytochrome a_3

$$\mathrm{cyt\ a_{3,o,n}} = \mathrm{[CCO]_{mit}} - \mathrm{cyt\ a_{3,r,n}} = 0.03201\,\mathrm{mM} \tag{151}$$

normal oxidised cytochrome a_3

$$\mathrm{cyt\ a_{3,r,init}} = \mathrm{cyt\ a_{3,r,n}} = 0.000828\,\mathrm{mM} \tag{152}$$

initial reduced cytochrome a_3

$$\mathrm{cyt\ a_{3,r,n}} = \frac{f_n\left(1 + \exp\left(-c3\left(\Delta\mathrm{p_n} - \Delta\mathrm{p_{30}}\right)\right)\right)}{k_3\mathrm{[O_2]_n}\exp\left(-c3\left(\Delta\mathrm{p_n} - \Delta\mathrm{p_{30}}\right)\right)} = 0.000828\,\mathrm{mM} \tag{153}$$

normal reduced cytochrome a_3

$$C_{\mathrm{NADH},n} = \frac{Z}{2}\log\left(\frac{1}{\mathrm{[NAD]_n/[NADH]_n}}\right) = -28.16\,\mathrm{mV} \tag{154}$$

normal value of C_{NADH}

$$\mathrm{[Cr]_n} = \frac{\mathrm{K^*_{eq,PCr}[ADP]_n[PCr]_n}}{\mathrm{[ATP]_n}} = 4.316\,\mathrm{mM} \tag{155}$$

normal concentration of creatine in cytoplasm

$$\mathrm{[CCO]_{mit}} = \frac{\mathrm{[CCO]_{tis}}}{V_{\mathrm{mit}}} = 0.03284\,\mathrm{mM} \tag{156}$$

concentration of cytochrome c oxidase in mitochondria

$$d_f = d\Delta_{\mathrm{nadir}} = 0\,(\mathrm{dimensionless}) \tag{157}$$

the fraction of cells which are considered as dead

$$\Delta G_{1,n} = -4\left(E_{1,n} + Z\log\left(\frac{\mathrm{Cu_{A,o,n}}}{\mathrm{Cu_{A,r,n}}}\right)\right) + p_1\Delta\mathrm{p_n} = -474.4\,\mathrm{mV} \tag{158}$$

normal free energy associated with reaction ared

$$\Delta G_{2,n} = -4\left(E_2 + Z\left(\log\left(\frac{\mathrm{Cu_{A,r,n}}}{\mathrm{Cu_{A,o,n}}}\right) - \log\left(\frac{\mathrm{cyt\ a_{3,r,n}}}{\mathrm{cyt\ a_{3,o,n}}}\right)\right)\right) + p_2\Delta\mathrm{p_n} \\ = -39.7\,\mathrm{mV} \tag{159}$$

normal free energy associated with reaction aox

$$\Delta G_n = \Delta G^\circ + ZF \log\left(g_{p,n}\right) = -5.91e + 04\,\mathrm{J\,mol^{-1}} \tag{160}$$

normal Gibbs free energy of ATP hydrolysis

$$D_{O2} = \frac{J_{O2,n}}{[O_{2,c}]_n - [O_2]_n} = 0.6796\,\mathrm{s^{-1}} \tag{161}$$

diffusion rate between capillaries and mitochondria

$$\Delta p_{30} = \Delta p_n + \Delta p_{3,corr} = 143.6\,\mathrm{mV} \tag{162}$$

value of PMF at which reaction box is maximally sensitive to Δp

$$\Delta p_{CV0} = \frac{-\Delta G_n}{n_a F} = 141.5\,\mathrm{mV} \tag{163}$$

a constant in the rate of Complex V

$$\Delta pH_n = pH_{m,n} - pH_{o,n} = 0.4\,(\text{dimensionless}) \tag{164}$$

the resting value of pH difference across mitochondrial inner membrane

$$\Delta p_n = \Delta\Psi_n + Z\Delta pH_n = 168.6\,\mathrm{mV} \tag{165}$$

the resting value of the proton motive force

$$\Delta\Psi_{init} = \Delta\Psi_n = 145\,\mathrm{mV} \tag{166}$$

initial value of mitochondrial inner membrane potential

$$E_{1,n} = E_{1,NADH,n} = 538.8\,\mathrm{mV} \tag{167}$$

the normal value of E_1

$$E_{1,NADH,n} = \mathcal{E}_0(Cu_A) - \mathcal{E}_0(NADH) + C_{NADH,n} = 538.8\,\mathrm{mV} \tag{168}$$

normal value of $E_{1,NADH}$

$$E_2 = \mathcal{E}_0(\text{cyt a}_3) - \mathcal{E}_0(Cu_A) = 103\,\mathrm{mV} \tag{169}$$

the energy provided by transfer of four electrons from $Cu_{A,r}$ to to cyt $a_{3,o}$

$$\mathrm{EPP} = 2[ATP]_n + [ADP]_n + [PCr]_n + [P]_n = 5.964\,\mathrm{mM} \tag{170}$$

the total exhangeable phosphate pool

$$f_n = \frac{CMRO_{2,n}}{V_{mit}} = 0.2985\,\mathrm{mM\,s^{-1}} \tag{171}$$

normal resting rate of f_1 and f_2

$$G_0 = G_{0,n} r0^4 = 0.001196\,\mathrm{ml_{blood}\,ml_{brain}}^{-1}\,\mathrm{mmHg^{-1}\,s^{-1}} \tag{172}$$

conductance of the supplying artery compartment

$$G_{0,n} = G_{0,\mathrm{frac}} G_n = 0.001196\,\mathrm{ml_{blood}}\,\mathrm{ml_{brain}}^{-1}\,\mathrm{mmHg}^{-1}\,\mathrm{s}^{-1} \tag{173}$$

normal conductance of the supplying artery compartment

$$\mathrm{glyc_{a,n}} = \frac{[\mathrm{ADP}]_\mathrm{n}^2}{k_{m,\mathrm{glycA}}^2 + [\mathrm{ADP}]_\mathrm{n}^2} = 0.9615\,(\mathrm{dimensionless}) \tag{174}$$

term in the expression for normal glycolysis rate

$$\mathrm{glyc_{g,n}} = \frac{[\mathrm{gluc}]_\mathrm{n}}{k_{m,\mathrm{glycG}} + [\mathrm{gluc}]_\mathrm{n}} = 0.96\,(\mathrm{dimensionless}) \tag{175}$$

term in the expression for normal glycolysis rate

$$\mathrm{glyc_{p,n}} = \frac{[\mathrm{P}]_\mathrm{n}^2}{k_{m,\mathrm{glycP}}^2 + [\mathrm{P}]_\mathrm{n}^2} = 0.9615\,(\mathrm{dimensionless}) \tag{176}$$

term in the expression for normal glycolysis rate

$$G_n = \frac{\mathrm{CBF_n}}{P_{a,\mathrm{n}} - P_{vs}} \left(1 + \frac{1}{G_{0,\mathrm{frac}}} + \frac{1}{G_{\mathrm{VArat,n}}}\right) = 0.0002392\,\mathrm{ml_{blood}}\,\mathrm{ml_{brain}}^{-1}\,\mathrm{mmHg}^{-1}\,\mathrm{s}^{-1} \tag{177}$$

normal resistance of cerebral circulation

$$g_{p,\mathrm{n}} = \frac{[\mathrm{ADP}]_\mathrm{n}[\mathrm{P}]_\mathrm{n}}{1000[\mathrm{ATP}]_\mathrm{n}} = 9.524e-06\,(\mathrm{dimensionless}) \tag{178}$$

normal phosphorylation potential

$$G_{t,n} = \frac{G_n G_{0,n} G_v}{G_n G_{0,n} + G_n G_v + G_v G_{0,n}} = 0.0001649\,\mathrm{ml_{blood}}\,\mathrm{ml_{brain}}^{-1}\,\mathrm{mmHg}^{-1}\,\mathrm{s}^{-1} \tag{179}$$

normal value of the total conductance of all blood vessel compartments

$$G_v = G_{\mathrm{VArat,n}} G_n = 0.0009567\,\mathrm{ml_{blood}}\,\mathrm{ml_{brain}}^{-1}\,\mathrm{mmHg}^{-1}\,\mathrm{s}^{-1} \tag{180}$$

conductance of the veins

$$\mathrm{HbO_{2,n}} = 1000 \frac{V_{\mathrm{a,n}}[\mathrm{HbO_{2,a}}]_\mathrm{n} + V_{\mathrm{v,n}}[\mathrm{HbO_{2,v}}]_\mathrm{n}}{4} V_{\mathrm{blood,n}} = 26.89\,\mathrm{\mu M} \tag{181}$$

normal total oxygenated haemoglobin (NIRS)

$$\mathrm{Hbt_n} = 1000 \frac{\left(V_{\mathrm{a,n}} + V_{\mathrm{v,n}}\right)[\mathrm{Hbtot}]_\mathrm{n}}{4} V_{\mathrm{blood,n}} = 43.88\,\mathrm{\mu M} \tag{182}$$

normal total haemoglobin (NIRS)

$$\mathrm{HHb_n} = 1000\frac{V_{\mathrm{a,n}}[\mathrm{HHb_a}]_\mathrm{n} + V_{\mathrm{v,n}}[\mathrm{HHb_v}]_\mathrm{n}}{4}V_{\mathrm{blood,n}} = 16.99\,\mu\mathrm{M} \tag{183}$$

normal total deoxygenated haemoglobin (NIRS)

$$\mathrm{H^+_{m,init}} = \mathrm{H^+_{m,n}} = 3.981e-05\,\mathrm{mM} \tag{184}$$

initial hydrogen ion concentration in mitochondria

$$\mathrm{H^+_{m,n}} = 10^{3-\mathrm{pH_{m,n}}} = 3.981e-05\,\mathrm{mM} \tag{185}$$

normal hydrogen ion concentration in mitochondria

$$h_n = -r_n + \sqrt{{r_n}^2 + 2r_0h_0 + h_0h_0} = 0.00214\,\mathrm{cm} \tag{186}$$

normal wall thickness of cerebral vessels

$$[\mathrm{H^+}]_\mathrm{n} = 1000 \times 10^{-\mathrm{pH_{o,n}}} = 0.0001\,\mathrm{mM} \tag{187}$$

normal hydrogen ion concentration in the cytoplasm

$$J_{\mathrm{O2},n} = \mathrm{CMRO_{2,n}} = 0.02\,\mathrm{mM\,s^{-1}} \tag{188}$$

the resting rate of supply of oxygen to the mitochondria

$$\mathrm{k_{1,n}} = \frac{f_n}{\mathrm{Cu_{A,o,n}} - \frac{1}{\mathrm{Keq_{1,n}}}\mathrm{Cu_{A,r,n}}} = 13.7\,\mathrm{s^{-1}} \tag{189}$$

the value of $\mathrm{k_1}$ at normal $\Delta\mathrm{p}$ and [NADH]

$$\mathrm{k_{2,n}} = \frac{f_n}{\mathrm{Cu_{A,r,n}cyt\ a_{3,o,n}} - \frac{1}{\mathrm{Keq_{2,n}}}\mathrm{Cu_{A,o,n}cyt\ a_{3,r,n}}} = 2681\,\mathrm{mM^{-1}\,s^{-1}} \tag{190}$$

normal forward rate constant for reaction aox

$$k_3 = \frac{k_{3,0}\left(1 + \exp\left(-c3\left(0 - \Delta\mathrm{p_{30}}\right)\right)\right)}{\exp\left(-c3\left(0 - \Delta\mathrm{p_{30}}\right)\right)} = 2.5e+05\,\mathrm{mM^{-1}\,s^{-1}} \tag{191}$$

rate constant for reaction box

$$\mathrm{Keq_{ADPATP}} = \frac{k_{\mathrm{AK}}}{k^-_{\mathrm{AK}}} = 2.784\,(\text{dimensionless}) \tag{192}$$

equilibrium constant for the conversion of ADP to ATP and AMP

$$k_{CV} = \frac{-1}{\Delta\mathrm{p_n} - \Delta\mathrm{p_{CV0}}}\ln\left(\frac{1 - L_{CV,0}}{1 + r_{CV}L_{CV,0}}\right) = 0.05927\,\mathrm{mV^{-1}} \tag{193}$$

a parameter controlling the sensitivity of Complex V flux to driving force

$$\mathrm{Keq}_{1,\mathrm{n}} = 10^{\left(-1/Z\right)\left(p_1 \Delta \mathrm{p_n}/4 - E_{1,n}\right)} = 50.31\,(\text{dimensionless}) \tag{194}$$

normal equilibrium constant for reaction ared

$$\mathrm{Keq}_{2,\mathrm{n}} = 10^{\left(-1/Z\right)\left(p_2 \Delta \mathrm{p_n}/4 - E_2\right)} = 0.07735\,(\text{dimensionless}) \tag{195}$$

normal equilibrium constant for reaction aox

$$K_G = \frac{G_n}{{r_n}^4} = 1956\,\mathrm{ml_{blood}\,ml_{brain}}^{-1}\,\mathrm{mmHg}^{-1}\,\mathrm{s}^{-1}\,\mathrm{cm}^{-4} \tag{196}$$

constant of proportionality relating pressure drop to flow

$$k_m = [\mathrm{ATP}]_\mathrm{n} k_{m,\mathrm{ATP}} = 0.03\,\mathrm{mM} \tag{197}$$

k_m for ATP use

$$k_{m,\mathrm{glycA}} = k_{m,\mathrm{glycA,f}}[\mathrm{ADP}]_\mathrm{n} = 0.0024\,\mathrm{mM} \tag{198}$$

k_m for ADP in the caricature of glycolysis

$$k_{m,\mathrm{glycP}} = k_{m,\mathrm{glycP,f}}[\mathrm{P}]_\mathrm{n} = 0.1905\,\mathrm{mM} \tag{199}$$

k_m for inorganic phosphate in the caricature of glycolysis

$$k_{m,\mathrm{tcaN}} = k_{m,\mathrm{tcaN,f}}[\mathrm{NAD}]_\mathrm{n} = 1.62\,\mathrm{mM} \tag{200}$$

k_m for NAD in the TCA cycle

$$k_{m,\mathrm{tcaP}} = k_{m,\mathrm{tcaP,f}}[\mathrm{Py}]_\mathrm{n} = 0.0005\,\mathrm{mM} \tag{201}$$

k_m for pyruvate in the TCA cycle

$$k^-_{\mathrm{PCr}} = \frac{\ln(2)}{\left(\mathrm{K_{eq,PCr}}[\mathrm{PCr}]_\mathrm{n}[\mathrm{ADP}]_\mathrm{n} + [\mathrm{Cr}]_\mathrm{n} + [\mathrm{ATP}]_\mathrm{n}\right) t_{1/2,\mathrm{PCr}}} = 0.6691\,\mathrm{mM}^{-1}\,\mathrm{s}^{-1} \tag{202}$$

the backward rate of reaction for the reaction in which phosphocreatine combines with ADP to give creatine and ATP

$$k^-_{\mathrm{pl}} = t_{1/2,\mathrm{pl}} = 10\,\mathrm{mM}^{-1}\,\mathrm{s}^{-1} \tag{203}$$

rate of backwards reaction in the pyruvate lactate equilibrium

$$k_{\mathrm{PCr}} = \frac{\ln(2)}{\left([\mathrm{PCr}]_\mathrm{n}[\mathrm{ADP}]_\mathrm{n} + \dfrac{[\mathrm{Cr}]_\mathrm{n} + [\mathrm{ATP}]_\mathrm{n}}{\mathrm{K_{eq,PCr}}}\right) t_{1/2,\mathrm{PCr}}} = 1.111e+06\,\mathrm{mM}^{-2}\,\mathrm{s}^{-1} \tag{204}$$

the forward rate of reaction for the reaction in which phosphocreatine combines with ADP to give creatine and ATP

$$\mathrm{K_{eq,PCr}} = \frac{\mathrm{K^*_{eq,PCr}}}{[\mathrm{H^+}]_\mathrm{n}} = 1.66e+06\,\mathrm{mM}^{-1} \tag{205}$$

equilibrium of the reaction in which phosphocreatine combines with ADP to give creatine and ATP

$$k_{\mathrm{TCA_n}} = \frac{1}{3}\frac{\mathrm{CMRO_{2,n}}}{V_{\mathrm{mit}}} = 0.0995\,\mathrm{mM\,s^{-1}} \tag{206}$$

normal rate of the TCA cycle

$$L_{CV,\mathrm{frac}} = 1 - L_{lk,\mathrm{frac}} = 0.75\,(\mathrm{dimensionless}) \tag{207}$$

normal fraction of proton entry into mitochondria associated with ADP phosphorylation

$$L_{CV,\mathrm{max}} = \frac{L_{CV,\mathrm{n}}}{L_{CV,0}} = 10.3\,\mathrm{mM\,s^{-1}} \tag{208}$$

the maximum rate of proton flow through Complex V

$$L_{CV,\mathrm{n}} = L_n L_{CV,\mathrm{frac}} = 4.119\,\mathrm{mM\,s^{-1}} \tag{209}$$

the resting flow of protons into the matrix through Complex V

$$L_{lk0} = \frac{L_{lk,\mathrm{n}}}{\exp\left(\Delta \mathrm{p_n} k_{lk2}\right) - 1} = 0.002269\,\mathrm{mM\,s^{-1}} \tag{210}$$

first constant controlling rate of L_{lk} of $\Delta\mathrm{p}$

$$L_{lk,\mathrm{n}} = L_n L_{lk,\mathrm{frac}} = 1.373\,\mathrm{mM\,s^{-1}} \tag{211}$$

the resting flow of protons into the matrix via leak channels

$$L_n = p_{tot} f_n = 5.492\,\mathrm{mM\,s^{-1}} \tag{212}$$

the total flow of protons back into mitochondria

$$\mathrm{NADH_n} = \mathrm{N_t} - [\mathrm{NAD}]_\mathrm{n} = 0.3\,\mathrm{mM} \tag{213}$$

normal concentration of NADH in the mitochondria

$$[\mathrm{NAD}]_\mathrm{n} = \frac{\mathrm{N_t}}{1 + \dfrac{1}{[\mathrm{NAD}]_\mathrm{n}/[\mathrm{NADH}]_\mathrm{n}}} = 2.7\,\mathrm{mM} \tag{214}$$

normal concentration of NAD in the mitochondria

$$[\mathrm{O_{2,a}}] = \phi \left(\frac{\mathrm{SaO_2}}{1 - \mathrm{SaO_2}}\right)^{1/n_h} = 0.1283\,\mathrm{mM} \tag{215}$$

arterial dissolved oxygen concentration

$$[\mathrm{O_{2,c}}]_\mathrm{init} = [\mathrm{O_{2,c}}]_\mathrm{n} = 0.05343\,\mathrm{mM} \tag{216}$$

initial concentration of dissolved oxygen in the capillary

$$[\mathrm{O_{2,c}}]_\mathrm{n} = \phi \left(\frac{\mathrm{ScO_{2,n}}}{1 - \mathrm{ScO_{2,n}}} \right)^{1/n_h} = 0.05343\,\mathrm{mM} \tag{217}$$

normal concentration of dissolved oxygen in the capillary

$$[\mathrm{O_2}]_\mathrm{init} = [\mathrm{O_2}]_\mathrm{n} = 0.024\,\mathrm{mM} \tag{218}$$

initial oxygen concentration in mitochondria

$$p_1 = p_{tot} - p_{23} = 10.4\,(\mathrm{dimensionless}) \tag{219}$$

the number of protons pumped by reaction ared

$$P_{1,n} = \frac{P_{a2,\mathrm{n}} + P_{v,\mathrm{n}}}{2} = 26.59\,\mathrm{mmHg} \tag{220}$$

normal average blood pressure in vessels

$$p_2 = 4 \times 1 = 4\,(\mathrm{dimensionless}) \tag{221}$$

total protons pumped by reaction aox

$$p_{23} = 4 \times 2 = 8\,(\mathrm{dimensionless}) \tag{222}$$

total protons pumped by reactions aox and box

$$p_3 = p_{23} - p_2 = 4\,(\mathrm{dimensionless}) \tag{223}$$

total protons pumped by reaction box

$$P_{a2,\mathrm{n}} = \frac{G_{0,n} P_{a,\mathrm{n}} + G_n P_{v,\mathrm{n}}}{G_n + G_{0,n}} = 43.31\,\mathrm{mmHg} \tag{224}$$

normal arterial blood pressure at the start of the cerebral arteries

$$\mathrm{PaO_2} = \frac{[\mathrm{O_{2,a}}]}{\mathrm{k_{H,O_2}}} = 91.68\,\mathrm{mmHg} \tag{225}$$

partial pressure of oxygen in the arteries

$$[\mathrm{P}]_\mathrm{n} = \frac{[\mathrm{PCr}]_\mathrm{n}}{[\mathrm{PCr}]_\mathrm{n}/[\mathrm{P_i}]_\mathrm{n}} = 0.9524\,\mathrm{mM} \tag{226}$$

the normal concentration of inorganic phosphate in the cytoplasm

$$P_{v,\mathrm{n}} = \frac{G_{t,n}}{G_v} \left(P_{a,\mathrm{n}} - P_{vs} \right) + P_{vs} = 9.862\,\mathrm{mmHg} \tag{227}$$

normal venous blood pressure

$$r0 = 1 - r_\mathrm{frac} r_\mathrm{occ} = 1\,(\mathrm{dimensionless}) \tag{228}$$

radius of the supplying artery compartment

$$r_{\mathrm{frac}} = 1 - (1 - \mathrm{occ}_{\mathrm{frac}})^{1/4} = 0.3313\,(\mathrm{dimensionless}) \tag{229}$$

fraction by which the radius of the supplying artery compartment is reduced if the carotid arteries are fully occluded

$$\mathrm{ScO}_{2,\mathrm{n}} = \frac{\mathrm{SaO}_{2,\mathrm{n}} + \mathrm{SvO2}_{\mathrm{n}}}{2} = 0.7285\,(\mathrm{dimensionless}) \tag{230}$$

normal capillary oxygen saturation

$$\sigma_{e,n} = \sigma_{e0}\left(\exp\left(\frac{K_\sigma\,(r_n - r_0)}{r_0}\right) - 1\right) - \sigma_{\mathrm{coll}} = -44.89\,\mathrm{mmHg} \tag{231}$$

normal elastic stress in vessel walls

$$\mathrm{SvO2}_{\mathrm{n}} = \frac{[\mathrm{HbO}_{2,\mathrm{v}}]_{\mathrm{n}}}{[\mathrm{Hbtot}]_{\mathrm{n}}} = 0.497\,(\mathrm{dimensionless}) \tag{232}$$

normal venous oxygen saturation

$$T_{e,\mathrm{n}} = \sigma_{e,n} h_n = -0.09604\,\mathrm{mmHg\,cm} \tag{233}$$

normal elastic tension in vessel walls

$$T_{\mathrm{max0}} = \frac{T_{\mathrm{max,n}}}{1 + k_{\mathrm{aut}}\mu_n} = 1.206\,\mathrm{mmHg\,cm} \tag{234}$$

T_{max} at normal μ

$$T_{\mathrm{max,n}} = \frac{T_{m,\mathrm{n}}}{\exp\left(-\left(\left|\frac{r_n - r_m}{r_t - r_m}\right|\right)^{n_m}\right)} = 1.206\,\mathrm{mmHg\,cm} \tag{235}$$

normal maximum muscular tension developed by circulation

$$T_{m,\mathrm{n}} = \left(P_{1,n} - P_{ic,\mathrm{n}}\right) r_n - T_{e,\mathrm{n}} = 0.5091\,\mathrm{mmHg\,cm} \tag{236}$$

normal muscular tension in vessel walls

$$u = u_n = 1\,(\mathrm{dimensionless}) \tag{237}$$

the representation of “demand” in the model

$$\nu_{\mathrm{CO}_2,n} = P_a\mathrm{CO}_{2,\mathrm{n}} = 40\,\mathrm{mmHg} \tag{238}$$

normal value of ν_{CO_2} ($P_a\mathrm{CO}_2$ passed through a first order filter)

$$\mathrm{V}_{\mathrm{glucosein}} = \frac{[\mathrm{gluc_c}]}{k_{\mathrm{glut}} + [\mathrm{gluc_c}]} = 0.4609\,(\mathrm{dimensionless}) \tag{239}$$

rate term for glucose transported into the cell; this is a michaelis menten term, but fixed because blood glucose concentration is fixed

$$v_{\mathrm{glut}} = \frac{\mathrm{CMR}_{\mathrm{gluc,n}}}{\mathrm{V}_{\mathrm{glucosein}} - \frac{[\mathrm{gluc}]_{\mathrm{n}}}{[\mathrm{gluc}]_{\mathrm{n}} + k_{\mathrm{glut}}}} = 0.01473\,\mathrm{mM\,s^{-1}} \quad (240)$$

Vmax for glucose transport both in and out of the cell from the capillary

$$\mathrm{V}_{\mathrm{lacin}} = \frac{[\mathrm{lac_c}][\mathrm{H^+}]_{\mathrm{n}}}{\left(k_{\mathrm{MCT}} + [\mathrm{lac_c}]\right)\left([\mathrm{H^+}]_{\mathrm{n}} + k_{\mathrm{MCT,H^+}}\right)} = 0.3333\,(\mathrm{dimensionless}) \quad (241)$$

rate of lactate transport into the cell

$$v_{\mathrm{MCT}} = \begin{cases} v_{\mathrm{MCT,val}} & \text{if } v_{\mathrm{MCT,val}} > 0 \\ \mathrm{NaN} & \text{otherwise} \end{cases} = 0.008\,\mathrm{mM\,s^{-1}} \quad (242)$$

rate constant for lactate transport

$$v_{\mathrm{MCT,val}} = \frac{2\mathrm{CMR}_{\mathrm{gluc,n}} - \frac{\mathrm{CMRO}_{2,\mathrm{n}}}{3}}{\frac{[\mathrm{lac}]_{\mathrm{n}}[\mathrm{H^+}]_{\mathrm{n}}}{\left([\mathrm{lac}]_{\mathrm{n}} + k_{\mathrm{MCT}}\right)\left([\mathrm{H^+}]_{\mathrm{n}} + k_{\mathrm{MCT,H^+}}\right)} - \mathrm{V}_{\mathrm{lacin}}} = 0.008\,\mathrm{mM\,s^{-1}} \quad (243)$$

Calculated value for v_{MCT}. If this is not greater than zero, parameter set is invalid.

$$v_{\mathrm{glyc,n}} = \frac{\mathrm{CMR}_{\mathrm{gluc,n}}}{\mathrm{glyc}_{\mathrm{g,n}}\mathrm{glyc}_{\mathrm{p,n}}\mathrm{glyc}_{\mathrm{a,n}}} = 0.004957\,\mathrm{mM\,s^{-1}} \quad (244)$$

normal Vmax for glycolysis which is assumed to be modified by the ratio of AMP to ATP

$$V_{\mathrm{a,n}} = \frac{V_{\mathrm{t,n}}}{1 + \mathrm{VArat_n}} = 0.25\,(\mathrm{dimensionless}) \quad (245)$$

normal arterial blood volume as a fraction of total blood volume

$$V_{\mathrm{v,n}} = V_{\mathrm{t,n}}\frac{\mathrm{VArat_n}}{1 + \mathrm{VArat_n}} = 0.75\,(\mathrm{dimensionless}) \quad (246)$$

normal venous volume

$$\nu_{\mathrm{O}_2,n} = [\mathrm{O}_{2,\mathrm{c}}]_{\mathrm{n}} = 0.05343\,\mathrm{mM} \quad (247)$$

normal value of ν_{O_2} ($[\mathrm{O}_{2,\mathrm{c}}]$ passed through a first order filter)

$$\nu_{P_a,n} = P_{a2,\mathrm{n}} = 43.31\,\mathrm{mmHg} \quad (248)$$

normal value of ν_{P_a} (P_{a2} passed through a first order filter)

$$v_{\mathrm{TCA}} = \frac{k_{\mathrm{TCA_n}}}{[\mathrm{Py}]_{\mathrm{n}}[\mathrm{NAD}]_{\mathrm{n}}}\left(k_{m,\mathrm{tcaN}} + [\mathrm{NAD}]_{\mathrm{n}}\right)\left(k_{m,\mathrm{tcaP}} + [\mathrm{Py}]_{\mathrm{n}}\right) = 0.16\,\mathrm{mM\,s^{-1}} \quad (249)$$

Vmax for the TCA cycle

$$\nu_{u,n} = u_n = 1\,(\text{dimensionless}) \tag{250}$$

normal value of ν_u (u passed through a first order filter)

$$[\mathrm{HHb_a}] = [\mathrm{Hbtot}]\,(1 - \mathrm{SaO_2}) = 0.216\,\mathrm{mM} \tag{251}$$

unsaturated arterial haemoglobin

$$[\mathrm{HHb_a}]_\mathrm{n} = [\mathrm{Hbtot}]_\mathrm{n}\left(1 - \mathrm{SaO_{2,n}}\right) = 0.216\,\mathrm{mM} \tag{252}$$

normal unsaturated arterial haemoglobin

$$[\mathrm{HbO_{2,a}}] = [\mathrm{Hbtot}]\mathrm{SaO_2} = 5.184\,\mathrm{mM} \tag{253}$$

concentration of arterial oxyhaemoglobin

$$[\mathrm{HbO_{2,a}}]_\mathrm{n} = [\mathrm{Hbtot}]_\mathrm{n}\mathrm{SaO_{2,n}} = 5.184\,\mathrm{mM} \tag{254}$$

normal concentration of arterial oxyhaemoglobin

$$[\mathrm{HbO_{2,v}}]_\mathrm{init} = [\mathrm{HbO_{2,v}}]_\mathrm{n} = 2.684\,\mathrm{mM} \tag{255}$$

initial concentration of venous oxyhaemoglobin

$$[\mathrm{HbO_{2,v}}]_\mathrm{n} = \frac{\mathrm{CBF_n}[\mathrm{HbO_{2,a}}]_\mathrm{n} - J_{\mathrm{O2},n}}{\mathrm{CBF_n}} = 2.684\,\mathrm{mM} \tag{256}$$

normal concentration of venous oxyhaemoglobin

$$[\mathrm{Hbtot}] = [\mathrm{Hbtot}]_\mathrm{n} = 5.4\,\mathrm{mM} \tag{257}$$

total haemoglobin concentration in the arteries and veins

$$[\mathrm{HHb_v}]_\mathrm{n} = [\mathrm{Hbtot}]_\mathrm{n} - [\mathrm{HbO_{2,v}}]_\mathrm{n} = 2.716\,\mathrm{mM} \tag{258}$$

normal unsaturated venous haemoglobin

References

[1] Roth, K. & Weiner, M. W., 1991 Determination of cytosolic ADP and AMP concentrations and the free energy of ATP hydrolysis in human muscle and brain tissues with 31-P NMR spectroscopy. *Magn. Reson. Med.* 22, 505–511. doi:10.1002/mrm.1910220258

[2] Corbett, R. J., Laptook, A. R., Garcia, D. & Ruley, J. I., 1993 Energy reserves and utilization rates in developing brain measured in vivo by 31P and 1H nuclear magnetic resonance spectroscopy. *J. Cereb. Blood Flow Metab.* 13, 235–246. doi:doi:10.1038/jcbfm.1993.29

[3] Flecknell, P. A., Wootton, R. & John, M., 1983 Cerebral blood flow and cerebral metabolism in normal and intrauterine growth retarded neonatal piglets. *Clin. Sci.* 64, 161–165

[4] Springett, R., Newman, J., Cope, M. & Delpy, D. T., 2000 Oxygen dependency and precision of cytochrome oxidase signal from full spectral NIRS of the piglet brain. *Am J. Physiol. Heart Physiol.* 279, H2202–H2209

[5] Aubert, A. & Costalat, R., 2001 Modelling of the coupling between brain electrical activity and metabolism. *Acta Biotheor.* 49, 301–326

[6] Ursino, M. & Lodi, C. A., 1998 Interaction among autoregulation, CO_2 reactivity, and intracranial pressure: a mathematical model. *Am. J. Physiol.* 274, H1715–H1728

[7] Korzeniewski, B. & Zoladz, J. A., 2001 A model of oxidative phosphorylation in mammalian skeletal muscle. *Biophys. Chem.* 92, 17–34

[8] Banaji, M., Tachtsidis, I., Delpy, D. & Baigent, S., 2005 A physiological model of cerebral blood flow control. *Math. Biosci.* 194, 125–173. doi:10.1016/j.mbs.2004.10.005

[9] Gruetter, R., Novotny, E. J., Boulware, S. D., Rothman, D. L., Mason, G. F., Shulman, G. I., Shulman, R. G. & Tamborlane, W. V., 1992 Direct measurement of brain glucose concentrations in humans by 13C NMR spectroscopy. *Proc. Natl. Acad. Sci. U. S. A.* 89, 1109–1112. doi:doi:10.1073/pnas.89.3.1109

[10] Edvinsson, L., Mackenzie, E. T. & Mcculloch, J., 1992 *Cerebral Blood Flow and Metabolism.* Raven Pr

[11] Lawson, J. W. & Veech, R. L., 1979 Effects of pH and free Mg^{2+} on the K_{eq} of the creatine kinase reaction and other phosphate hydrolyses and phosphate transfer reactions. *J. Biol. Chem.* 254, 6528–6537

[12] Rosenkrantz, T. S., Kubin, J., Mishra, O. P., Smith, D. & Delivoria-Papadopoulos, M., 1996 Brain cell membrane Na^+, K^+-ATPase activity following severe hypoxic injury in the newborn piglet. *Brain Res.* 730, 52–57

[13] Brand, M., Pakay, J. L., Ocloo, A., Kokoszka, J., Wallace, D., Brookes, P. & Cornwall, E., 2005 The basal proton conductance of mitochondria depends on adenine nucleotide translocase content. *Biochem. J.* 392, 353. doi:10.1042/BJ20050890

[14] Pourcyrous, M., Leffler, C. W., Bada, H. S., Korones, S. B. & Busija, D. W., 1994 Cerebral blood flow responses to indomethacin in awake newborn pigs. *Pediatr. Res.* 35, 565–570. doi:doi:10.1203/00006450-199405000-00007

[15] Erecinska, M., Cherian, S. & Silver, I. A., 2004 Energy metabolism in mammalian brain during development. *Prog. Neurobiol.* 73, 397–445. doi:10.1016/j.pneurobio.2004.06.003
